# New Acetophenones and Chromenes from the Leaves of *Melicope barbigera* A. Gray

**DOI:** 10.3390/molecules26030688

**Published:** 2021-01-28

**Authors:** Kim-Thao Le, Jan J. Bandolik, Matthias U. Kassack, Kenneth R. Wood, Claudia Paetzold, Marc S. Appelhans, Claus M. Passreiter

**Affiliations:** 1 Institute of Pharmaceutical Biology and Biotechnology, Heinrich-Heine-University Duesseldorf, 40225 Duesseldorf, Germany; kim.le@hhu.de; 2 Institute for Pharmaceutical and Medicinal Chemistry, Heinrich-Heine-University Duesseldorf, 40225 Duesseldorf, Germany; jan.bandolik@hhu.de (J.J.B.); matthias.kassack@hhu.de (M.U.K.); 3 National Tropical Botanical Garden, 3530 Papalina Road, Kalaheo, HI 96741, USA; kwood@ntbg.org; 4 Institute of Systematics, Biodiversity and Evolution of Plants, Georg-August-University Goettingen, 37073 Goettingen, Germany; claudia.paetzold@biologie.uni-goettingen.de (C.P.); marc.appelhans@biologie.uni-goettingen.de (M.S.A.); 5 Division Botany and Molecular Evolution, Senckenberg Gesellschaft für Naturforschung, Senckenberganlage 25, 60325 Frankfurt am Main, Germany

**Keywords:** *Melicope barbigera*, Rutaceae, acetophenones, chromenes, melibarbinon A and B, melibarbichromen A and B, cytotoxicity, ovarian cancer cell line A2780

## Abstract

The dichloromethane extract from leaves of *Melicope barbigera* (Rutaceae), endemic to the Hawaiian island of Kaua’i, yielded four new and three previously known acetophenones and 2*H*-chromenes, all found for the first time in *M. barbigera*. The structures of the new compounds obtained from the dichloromethane extract after purification by chromatographic methods were unambiguously elucidated by spectroscopic analyses including 1D/2D NMR spectroscopy and HRESIMS. The absolute configuration was determined by modified Mosher’s method. Compounds **2**, **4** and the mixture of **6** and **7** exhibited moderate cytotoxic activities against the human ovarian cancer cell line A2780 with IC_50_ values of 30.0 and 75.7 µM for **2** and **4**, respectively, in a nuclear shrinkage cytotoxicity assay.

## 1. Introduction

The genus Melicope J.R. Forst. and G. Forst. is a member of the Rutaceae (Rue or Citrus family) and contains circa 239 species distributed in the Malagasy, Indo-Himalayan, South-East Asian, and Pacific regions [1,2]. With 54 currently accepted endemic species on the Hawaiian Islands, *Melicope* ranks among the three most speciose lineages of the archipelago [3,4]. Among Hawaiian *Melicope*, several species are endangered or even considered to be extinct [3,5,6]. However, some new Hawaiian species have recently been discovered and botanically described [6,7,8]. *Melicope* has been subdivided into four sections based on morphology, and molecular phylogenetic studies have demonstrated that only one of them is monophyletic [1,2]. All Hawaiian species belong to section Pelea. Rutaceae are known for their extremely diverse secondary metabolites that include many alkaloids derived from anthranilic acid, limonoids, coumarins, and acetophenones. *Melicope* species are proven to be producers of many interesting secondary metabolites including polymethoxylated flavonoids, furanocoumarins, acetophenones and quinolone alkaloids [9,10,11,12,13,14]. Moreover, several *Melicope* species are used in traditional and modern medicine [15,16,17]. Since some of the compounds isolated from *Melicope* possessed antibacterial, antidiabetic, cytotoxic and antiproliferative activities in human cancer cell lines [15], *Melicope* species are of special interest for the continuation of our cytotoxicity studies from various plants [18,19].

While the phytochemistry of some species is sufficiently characterized, the information for many species, especially those from Pacific Islands and rare species with narrow distributions, is rather scarce. Because of the threat of extinction, it seems especially important to chemically characterize the species endemic to the Hawaiian archipelago. In addition to morphological and genomic characters, the pattern of secondary metabolites could also serve as a third source of information to distinguish between morphologically similar species or populations within a species. In the current study, we focus on *Melicope barbigera*, which is an example of an understudied species with a very narrow distribution range, limited to mesic forests in North-Western Kaua’i. This species was only chemically investigated in a single study from 1974, which reported the isolation of four coumarins and two highly methylated flavones, which are characteristic for the genus *Melicope* [20].

**Figure 1:**
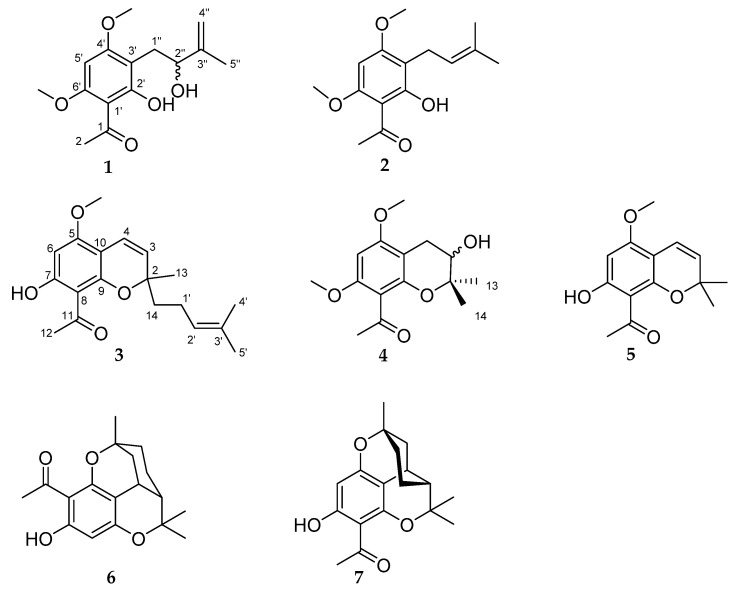
Structures of compounds **1**−**7** isolated from the leaves of *Melicope barbigera.*

We concentrated on the screening of the dichloromethane extract obtained from ground leaves of *M. barbigera*. After purification, using various chromatographic methods, we isolated the acetophenones **1** and **2** as well as the 2*H*-benzopyranes (chromenes) **3** and **4**, all four found for the first time in nature, in addition to the known chromenes alloevodionol (**5**) [21] and the isomeric melifoliones **6** and **7** [22], only isolated as mixture (2.5:1) (see Figure 1). The pure compounds **1**–**5** and the mixture of **6** and **7** were tested for their cytotoxic activities against the human ovarian cancer cell line A2780. Compounds **2** and **4** exhibited moderate cytotoxic activities with IC_50_ values of 30.0 μM and 75.7 μM, respectively.

## 2. Results and Discussion

**Figure 2:**
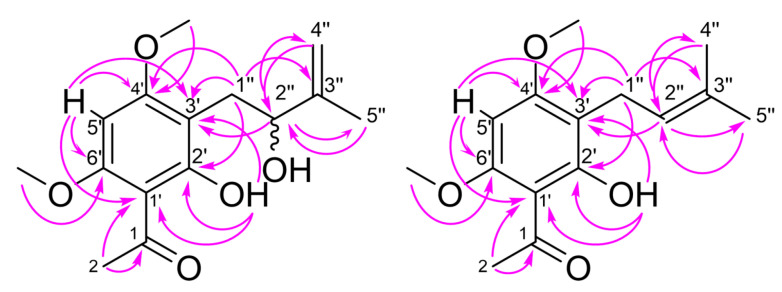
Key correlations of **1** and **2** in the HMBC spectrum.

Compound **1** was isolated as a yellowish-brown oil. Its molecular formula was determined as C_15_H_20_O_5_ by high resolution electrospray ionization mass spectrometry (HRESIMS), requiring six degrees of unsaturation. The ^13^C NMR spectrum of **1** (Table 1) displayed the signals of fifteen carbons, eight of which found to be protonated by their proton-carbon correlation in the two-dimensional Heteronuclear Single Quantum Correlation spectrum (HSQC). Six carbons were detected at shift values characteristic for a phenolic ring system, bearing four substituents in addition to the phenolic hydroxyl group located at C-2′ (*δ* 163.1). Its proton signal was found at *δ* 13.98 (s, 2′-OH) in the corresponding ^1^H NMR spectrum (see Table 1), indicating the formation of an intramolecular hydrogen bond with a nearby carbonyl group. Due to the downfield shift and the correlations with three of the benzene carbons found in the two-dimensional Heteronuclear Multiple Bond Correlation spectrum (HMBC), an acetyl substituent, and a hydroxylated prenyl side chain were found to be ortho to the phenolic hydroxyl group. The corresponding benzene carbons showed a correlation to a singlet at *δ* 6.21 (H-5′), which was assigned to an aromatic proton at C-5′ (*δ* 87.1), additionally correlated to two further benzene carbons at *δ* 55.8 (C-4′) and *δ* 55.9 (C-6′), each bearing a methoxy group. The hydroxylated benzene side chain was identified as a 2-hydoxy-3-methylbut-3-en-1-yl moiety, which was already found in other natural products. All assignments were additionally confirmed by their respective correlations in the 2D-COSY, HSQC, and HMBC spectra (see Figure 2). In order to determine the absolute configuration at the asymmetric carbon C-2′’, Mosher ester derivatives were prepared using a well-established method [23,24]. As a result of the reaction of **1** with the (*R*)- and (*S*)-Mosher reagents, we found NMR signals for mixtures of two diastereomeric ester derivatives, respectively. Compound **1** was therefore identified as a mixture of the enantiomeric (*R*)- and (*S*)- 1-(2-hydroxy-3-(2-hydroxy-3-methylbut-3-en-1-yl)-4,6-dimethoxyphenyl)ethan-1-one. Since the optical rotation [α]^20^_D_ was found to be minus 7.2° (see Materials and Methods), one of the enantiomers seems to be slightly higher concentrated. This could also be seen in the ^1^H NMR spectrum of the prepared Mosher ester derivatives, in which a difference of 10% was found for the integrals of the two diastereomers. Compound **1**, for which we propose the name melibarbinon A, was found for the first time in nature. However, a similar acetophenone was isolated from *Acronychia*, a genus of the Rutaceae closely related to *Melicope* [25,26].

The molecular formula of **2** (C_15_H_20_O_4_)**,** determined by HRESIMS analysis, indicated the loss of one oxygen atom compared to **1**. The ^13^C NMR spectrum of **2** also displayed 15 carbon signals at shift values similar to **1**. Differences were only found for the signals of the prenyl side chain and the aromatic carbon C-3′ (*δ* 108.5), where the side chain is attached. Instead of one methyl group, one unsaturated methylene and one aliphatic hydroxyl group, we found the signals for two methyl groups at *δ* 1.59 (s, H-4″) and *δ* 1.68 (s, H-5″) together with one proton at *δ* 5.07 (t, H-2″) indicating the presence of a 3-methyl-but-2-en-1-yl side chain, found in many prenylated natural products such as O-prenylated acetophenones from *M. obscura* and *M. obtusifolia* or the prenylated benzene pteleifolins A isolated from *M. pteleifolia* [27,28]. The structure was additionally confirmed by the correlations found in the 2D-NMR spectra (see Figure 2). This compound was also found for the first time in nature, but it was already described as intermediate in the synthesis of 4′-*O*-methylxanthohumol [29]. However, its completely assigned NMR data are given here for the first time. In analogy to **1**, we propose the name melibarbinon B for **2**.

**Figure 3:**
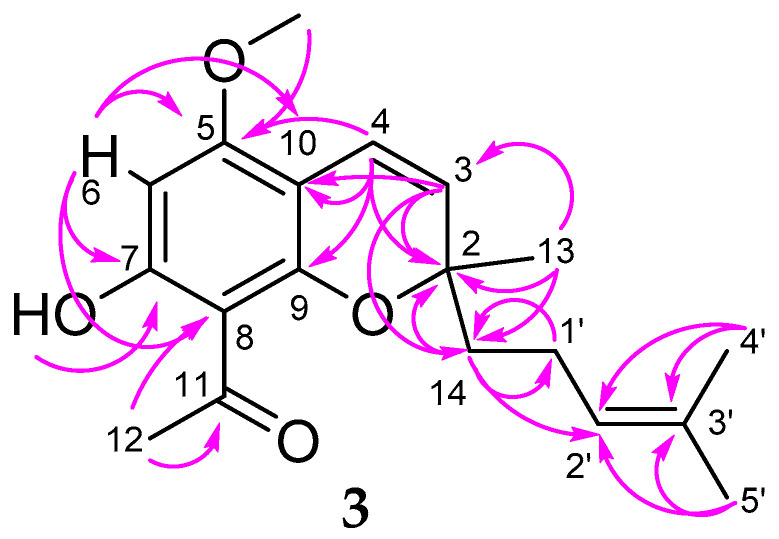
Key correlations of **3** in the HMBC spectrum.

Compound **3** was isolated as a yellow oil. The molecular formula was established as C_19_H_24_O_4_, indicating 6 degrees of unsaturation. The 1D-and 2D-NMR spectra of **3** (Table 2) showed the presence of a benzopyran moiety as found in alloevodionol (**5**) [21]. In comparison to the signals found in the spectra of **5**, the carbon signal of C-2 (*δ* 80.9) was slightly shifted upfield and the signal for two equivalent methyl carbons attached to C-2 in **5** was replaced by one methyl group (C-13, *δ* 26.7) and one methylene group (C-14, *δ* 41.7). This was confirmed by the ^3^J-Korrelation between H-3 (d, *δ* 5.38) of the chromene moiety in **3** with both methyl and methylene carbons. Thus, C-14 was found to be substituted by a prenyl side chain, showing typical proton and carbon shift values (Table 2). All signals were assigned by their correlations in the 2D-COSY, HMQC, and HMBC spectra of **3** (see Figure 3). The structure of **3** was found to be 1-[7-hydroxy-5-methoxy-2-methyl-2-(4-methylpent-3-en-1-yl)-2*H*-1-benzopyran-8-yl]ethan-1-one, previously described as intermediate in the chemical synthesis of boesenbergin A, a natural constituent of *Boesenbergia rotunda* (Zingiberaceae) [30]. However, **3** was found here for the first time in nature, so we suggest the name melibarbichromen A for this new natural compound.

**Figure 4:**
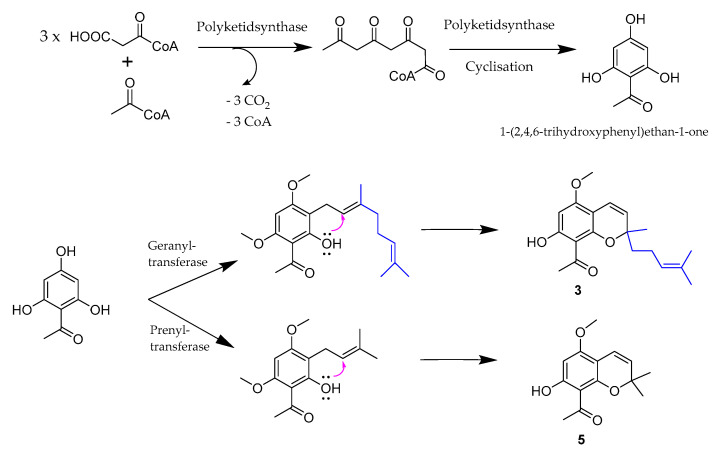
Proposed biosynthesis of chromenes **3** and **5**.

Compound **3** is possibly biosynthesized by reaction of an acetophenone with geranylpyrophosphate forming the pyrane ring and the exocyclic side chain, whereas **5** was formed by alkylation of a corresponding acetophenone with an unsaturated hemiterpene (see Figure 4). The assumption that the reaction of a hydroxylated acetophenone derivative with geranylpyrophosphate resulted in formation of a benzopyrane ring system was also made by Schmidt et al. who reported the obvious building of empetrifranzinan A and B in *Hypericum empetrifolium* (Hypericaceae) [31]. Both compounds are very similar to **6** and **7** isolated here, only differing by the presence of an isobutyl group instead of the acetyl group at C-6 and C-8 in **6** and **7**, respectively. Compound **6** and **7**, namely melifolione b and a, were already isolated as a 3:2 (a:b) mixture by Goh et al. from *Melicope latifolia* (Rutaceae; treated as *Euodia latifolia*) [22]. All attempts to separate **6** from **7** were not successful in our case. This was in accordance to the finding in the lab of Schmidt et al., where both empetrifranzinan derivatives were also isolated as a mixture [31]. However, Goh et al. were able to crystallize a small quantity of their main constituent melifolione a (**7**) in pure form, from which they obtained X-ray data confirming the stereochemistry of **7** [22]. Possibly, the purification of one of the two similar compounds failed because *M. barbigera* contained a different proportion of the two compounds with melifolione b (**6**) as main constituent.

**Figure 5:**
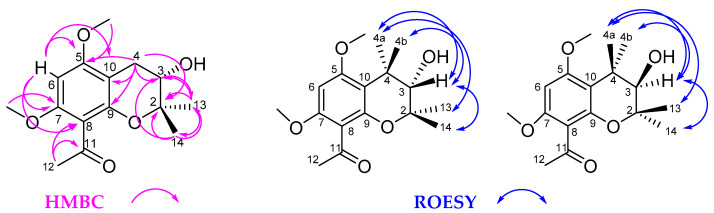
Key correlations of the racemic **4** in the HMBC and ROESY spectra.

The molecular formula of **4**, isolated as colorless oil, was determined as C_15_H_20_O_5_ by HRESIMS, suggesting six degrees of unsaturation. Interpretation of its ^1^H and ^13^C NMR spectra (Table 2) showed the presence of a 3,4-dihydro-benzopyrane ring system differing from **3** and **5** by the presence of two methoxy groups at *δ* 1.33 and 1.31 (s, H-13, H-14), respectively, and the absence of the double bond between C-3 and C-4. Instead of the two unsaturated carbons C-3 and C-4, the spectra clearly indicated the presence of one hydroxylated methine carbon (C-3) at *δ* 69.3 and one methylene carbon (C-4) at *δ* 26.2. The position of the hydroxy group at C-3 was clearly detected by correlations between C-3 and the ^1^H NMR signals of the two methyl groups at C-2 of the pyrane ring moiety. The assignment of the methoxy groups at C-5 unambiguously followed from correlations between H-4 and C-5 in the 2D-HMBC spectra (see Figure 5).

In order to assign the absolute configuration at C-3 of **4**, Mosher esters were prepared using the modified method of Su et al. [32]. Comparable to the reaction of **1** with the (*R*)-and (*S*)-Mosher reagents, the ^1^H NMR spectra in pyridine-*d*_5_ of the formed Mosher esters of **4** displayed the signals for a mixture of two diastereomeric esters in both cases. Compound **4** was therefore also identified as a racemic mixture of 1-[3,4-dihydro-3-hydroxy-5,7-dimethoxy-2,2-dimethyl-2*H*-benzopyran-8-yl]ethanone (see Materials and Methods). Due to the finding that **4** is a racemic mixture of the (*R*)-and (*S*)-enantiomers the position of the proton at C-3 is either α-or *β*-oriented to the benzopyrane moiety, respectively. However, the relative configuration could be detected from the contacts of this proton (*δ* 3.77 t, H-3) with the signal of one of the methyl groups at C-2 (*δ* 1.31 s, H-14). The latter signal showed a correlation to the proton at *δ* 2.84 (dd, H-4b), while the signal of the other methyl group (*δ* 1.33 s, H-13) showed a contact to the signal at *δ* 2.62 (dd, H-4a) in the Rotating frame Overhauser Enhancement Spectroscopy (ROESY) spectrum (see Figure 5).

Compound **4** was also not found in nature so far, to the best of our knowledge. We therefore propose the name melibarbichromen B. However, structurally similar compounds were already found in *Acronychia trifoliolata* and *Melicope pteleifolia* [9,33].

**Figure 6:**
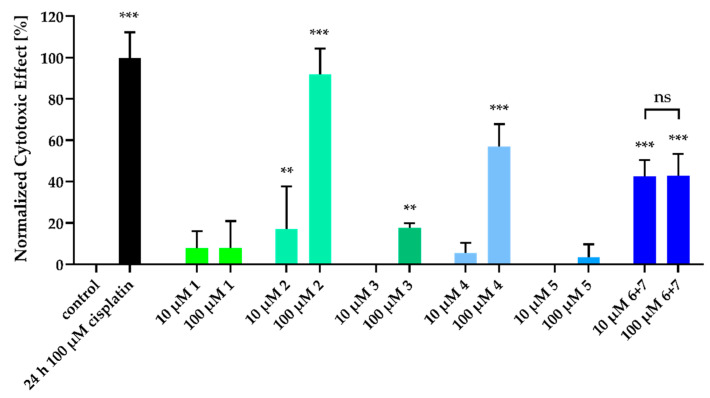
Cytotoxic activity of compounds of *Melicope barbigera.* A2780 cells were incubated with the compounds in the indicated concentrations for 72 h. Cell culture medium was added as a control for vehicle treated cells (“control”). A 24 h treatment with 100 µM cisplatin served as positive control. Data are the mean ± SD, *n* ≥ 3. Statistical analysis to compare the effects of compound and control was performed using t-test. For normalization, the value of the vehicle control was set to 0% and the 24 h 100 µM cisplatin control was set to 100%. Levels of significance: ns (*p* > 0.05); ** (*p* ≤ 0.01); *** (*p* ≤ 0.001) Effect bars without annotation are ns. Representative fluorescent imaging pictures for compounds with significant effects are shown in supplemental Figure S32.

**Figure 7:**
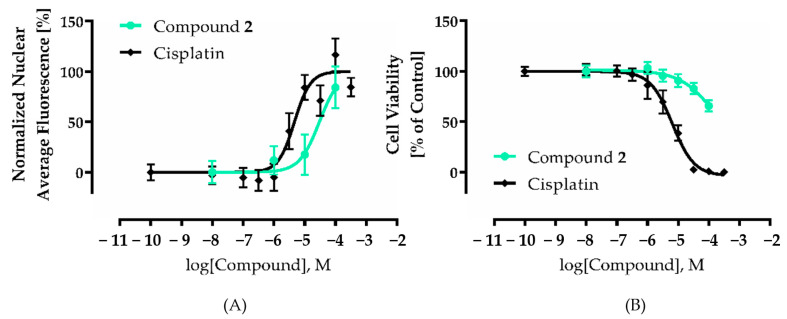
Cytotoxic activity of compound 2 in nuclear shrinkage assay and MTT assay. A2780 cells were incubated with compound **2** and cisplatin in the indicated concentrations for 72 h and effects were investigated with fluorescent based nuclear shrinkage assay (**A**) and MTT assay (**B**). Cell culture medium was added as a control for vehicle treated cells. For normalization of nuclear shrinkage assay effects, the value of the vehicle control was set to 0% and the 24 h 100 µM cisplatin control was set to 100% (**A**). A 24 h (**A**) or 72 h (**B**) treatment with 100 µM cisplatin served as positive control. The bottom value of the concentration effect curve of compound 2 in MTT assay was constrained to the effect of positive control (**B**). Data are the mean ± SD (**A**,**B**), *n* ≥ 3. IC_50_, pIC_50_, and SEM derived from four-parameter logistic equation are shown in Table 3.

We could demonstrate that the isolated acetophenones exhibited cytotoxicity against the human ovarian cancer cell line A2780 (see Figure 6 and Figure 7). This cell line was chosen because of our experience in screening natural products [34,35,36]. Interestingly, compounds **2** and **4** showed concentration-dependant cytotoxic effects in a nuclear shrinkage cytotoxicity assay, which were most pronounced for **2**. IC_50_ values were 30.0 µM for **2** (Table 3) and 75.7 µM for **4** (pIC_50_ ± SEM: 4.12 ± 0.18). Surprisingly, the mixture of **6** and **7** did not show concentration-dependant cytotoxicity so that no IC_50_ value could be derived. Nuclear shrinkage assays are used in the literature to detect morphological changes of the cell during apoptosis and resulting cell death, since apoptotic cells and their nuclei shrink during this process [37]. This is also explicitly described for natural products [38]. For the most potent compound (**2**) in the nuclear shrinkage assay (see Figure 5) we also performed MTT assays (72 h incubation period; Figure 6) to further characterize the cytotoxic effect. The IC_50_ value found for compound **2** in the MTT assay was higher than 100 µM (Table 3) and thus higher than the IC_50_ determined for **2** in the nuclear shrinkage assay, whereas cisplatin gave similar IC_50_ values in both tests (see Figure 6). Differences in the IC_50_ values of compound **2** in these two cytotoxicity assays may be attributed to the generally low cytotoxic effect of **2** and longer survival of mitochondria including mitochondrial dehydrogenases which are targeted by MTT assay reagent leading to earlier nuclear shrinkage than degradation of mitochondria. In conclusion, these data show moderate cytotoxic effects of compound **2** compared to cytotoxic agents like cisplatin. Since no compound showed a remarkably high cytotoxicity at a concentration of 10 µM, lower concentrations were not investigated. Thus, our findings confirm the results regarding cytotoxic activities of acetophenones [39,40].

Subsequently, we investigated if the cytotoxic effects shown in Figure 5 were caspase-dependent or not. Figure S33 shows the significant effect of the cytotoxic compounds **2** and **4** on the activation of caspase 3/7, which is essential in the induction and execution of apoptosis [41]. Taken together, our results are confirming the previously reported bioactivities of acetophenones [40,42,43,44]. Moreover, we also found that acetophenone derivatives containing prenyl substituents show particularly higher cytotoxic activities compared to other compounds not bearing such structure elements. This finding was also reported for species of the genus *Acronychia* [25,39,45,46,47] and we also observed such effects in our previous work with prenylated isoflavonoids and pterocarpanes from the genus *Erythrina* (Fabaceae) [48,49,50].

These findings demonstrate that endemic underexplored species such as *Melicope barbigera* are promising sources for deeper investigation. Moreover, enhanced research is needed to conserve the species and to obtain new sources for further natural product discovery. Acetophenones and chromenes have been discovered in 14 species of *Melicope* [11,14,15,16,17,27,51,52,53,54,55,56,57,58] as well as in some species of its close relatives *Acronychia* and *Medicosma* [33,46,59,60,61,62,63,64]. Due to the great variability of the acetophenones and chromenes in these genera, they may be of interest as biomarkers for chemotaxonomy. So far, leptonol and evodione have been found in two species: *M*. *lunu-ankenda* and *M. pteleifolia* [16,65]. The two species are close relatives and belong to *Melicope* section *Lepta* [3,10]. Highly similar xanthoxylin-derivates have been reported from three closely related *Melicope* species from Madagascar and the Mascarenes [17,64,66]. Characteristic prenylated acetophenones have also been reported for three *Acronychia* species. In *Acronychia*, two dimeric acetophenones were reported to have cytotoxic properties [47]. While acetophenones with geranyl substituents and compounds with an oxidized acetyl group have only been reported from *Melicope* so far [27], prenylated dimeric acetophenone derivatives are only known from *Acronychia* [67,68,69]. This could possibly mean that prenylated acetophenones can be regarded as chemotaxonomically informative at both the genus and the species level. The isolated acetophenones found in *M*. *barbigera* are most similar to those described from *M. pteleifolia* [69,70]. These two species are not close relatives within *Melicope*. However, *M*. *barbigera* belongs to section *Pelea* and no other species of that section so far has been tested for the presence of acetophenones. The occurrence of alloevodionol and its derivatives in at least five *Melicope* species belonging to three different sections as well as *Medicosma* [64,71,72], shows that some acetophenones and chromenes seem to be more ubiquitous in *Melicope* and related genera and are thus not chemotaxonomically informative. Chromenes and acetophenones found in *Melicope* generally are prenylated phloroglucin derivatives containing terpenoid side chains, e.g., isopentenyl-or geranyl-moieties. The pyrane ring in chromenes is subsequently build by intramolecular reaction of the unsaturated side chain in acetophenones with one ortho positioned hydroxyl group, from which a plausible biosynthetic pathway could be proposed [13,70]. A denser screening for acetophenones and chromenes in *Melicope* is needed in order to test their suitability as markers for chemotaxonomy. However, the variability of the compounds identified so far, is promising.

## 3. Materials and Methods

### 3.1. General Experimental Procedures

Optical rotations were measured on a Jasco P-2000 polarimeter (JASCO, Tokyo, Japan). NMR spectra were recorded on a Bruker ARX 300 or AVANCE DMX 600 NMR spectrometers (Bruker, Karlsruhe, Germany). Mass spectra were obtained from an Ion-Trap-API Finnigan LCQ Deca XP mass spectrometer while high resolution mass spectra were recorded on a FTHRMS-Orbitrap (Thermo-Finnigan, Waltham, MA, USA) mass spectrometer. A Dionex P580 system (Dionex Softron, Germering, Germany) was used in combination with a diode array detector UVD340S (Dionex Softron, Germering, Germany) and a Eurosphere 10 C_18_ column, 125 × 4 mm, (Knauer, Berlin, Germany). for HPLC analysis and UV spectra recording. Semi-preparative HPLC was conducted on a Lachrom-Merck Hitachi system (pump L7100, UV detector L7400, Eurosphere 100 C_18_ column, 300 × 8 mm (Knauer, Berlin, Germany)). Sephadex-LH20 and Merck MN silica gel 60 M (0.04–0.063 mm) were used as stationary phases for column chromatography. TLC was performed on silica gel 60 F_254_ plates sprayed with anise aldehyde/H_2_SO_4_ (VWR, Darmstadt, Germany) or 1% methanolic diphenylboryloxyethylamine (VWR; Darmstadt, Germany) and 5% methanolic polyethylene glycol 400 reagents (VWR, Darmstadt, Germany), respectively. For spectroscopic measurements spectral grade solvents were used. All other reagents met at least the analytical grade or at least HPLC grade for HPLC usage, respectively.

### 3.2. Plant Material

*Melicope barbigera* leaves (1 kg) were collected and identified in Kaua’i, Hawaii, USA, by Kenneth R. Wood, National Tropical Botanical Garden (NTBG) in Kalaheo, Kaua’i. A representative voucher specimen (PTBG1000062417) has been deposited at the NTBG herbarium and duplicates have been distributed (*Wood and Walsh 17238*, BISH, CAS, CAU, MBK, NY, PTBG, US).

### 3.3. Extraction and Isolation

Leaves were extracted using our standard method (Soxhlet, CH_2_Cl_2_) to give 95 g crude extract [19,48,50]. Purification was carried out by vacuum liquid chromatography (*n*-hexane/EtOAc and CH_2_Cl_2_/MeOH) to give 12 fractions (VLC I-XII). Further purification of the respective fractions conducting CC on Sephadex LH-20, silica gel and semi-preparative HPLC using a gradient of MeOH-H_2_O (0–1 min 30:70, 1–30 min to 100:0) gave 2.2 mg of **3** and 23 mg of **5** (VLC II), 2.4 mg of a mixture of **6** and **7** (VLC III) as well as 2.3 mg of **1**, 3.2 mg of **2** and 1.8 mg of **4** (VLC IV).

*1-(2-hydroxy-3-(2-hydroxy-3-methylbut-3-en-1-yl)-4,6-dimethoxyphenyl)ethan-1-one., melibarbinon A* (**1**): yellowish-brown oil; [α] ^20^_D_ -7.2 (*c* 0.2, MeOH); UV (MeOH) λ_max_ 214 nm and 292 nm; ^1^H and ^13^C NMR, Table 1; HRESIMS *m/z* 281.1384 [M + H]^+^ (C_15_H_21_O_5_, calcd. 281.1344).

*2-Hydroxy-4,6-dimethoxy-3-prenylacetophenone, melibarbinon B* (**2**): amorphous, white powder; UV (MeOH) λ_max_ 217 nm and 296 nm; ^1^H and ^13^C NMR data, Table 1; HRESIMS *m/z* 265.1435 [M + H]^+^ (C_15_H_21_O_4_, calcd. for 265.1395).

*1-[7-hydroxy-5-methoxy-2-methyl-2-(4-methylpent-3-en-1-yl)-2H-1-benzopyran-8-yl]ethan-1-one, melibarbichromen A* (**3**): yellow oil; [α]^20^_D_ -7.1 (*c* 0.2, MeOH); UV (MeOH) λ_max_ 221 nm; 283 nm; ^1^H and ^13^C NMR data, Table 2; HRESIMS *m/z* 317.1752 [M + H]^+^ (C_19_H_25_O_4_,calcd. for 317.1708).

*1-(3,4-dihydro-3-hydroxy-5,7-dimethoxy-2,2-dimethyl-2H-benzopyran-8-yl)ethenone, melibarbichromen B* (**4**): colourless oil; [α]^20^_D_-9.9 (*c* 0.2, MeOH); UV (MeOH) λ_max_ 208 nm and 281 nm; ^1^H and ^13^C NMR data, Table 2; ^1^H NMR in pyridine-*d_5_* see 3.4., HRESIMS *m/z* 281.1385 [M + H]^+^ (C_15_H_21_O_5_, calcd. for 281.1344).

### 3.4. Preparation of (R)-and (S)-MTPA Esters

The preparation of (*R*)-and (*S*)-MTPA esters of **1** and **4** were carried out using the method of Ohtani et al. [23]. Two samples of **1** (0.8 mg (0.0028 mmol)) and two samples of **4** (0.7 mg (0.0025 mmol)), respectively, were dissolved in 0.75mL pyridine-*d_5_* (VWR, Darmstadt, Germany) 10 µL of (*R*)-and (*S*)-MTPA chloride (α-methoxy-α(trifluoromethyl)phenylacetyl) chloride) reagent (VWR, Darmstadt, Germany) was added to all tubes and the reaction was equilibrated at room temperature (20 °C) for 8 h. All steps were performed under argon stream to avoid oxidation. ^1^H NMR spectra were recorded of the two sets after purification using semi-preparative HPLC (MeOH-H_2_O; 0–2 min 40:60, 2–20 min, 100:0).

(*R*)-MTPA ester of **1**: ^1^H NMR (Pyridin-*d_5_*): δ_H_ 14.76 and 14.69 (s, 2′-OH, 1:0.9), 6.16 and 6.04 (s, H-5′, 1:0.9), 5.16 and 4.97 (d, H-4′’-H, 1:0.9), 5.23 and 5.00 (dd, H-2′’, 0.9:1), 3.19 and 3.13 (dd, H-1′’,0.9:1), 3.60 (s, OCH_3_ at C-6′, overlapped), 3.74 and 3.73 (s, OCH_3_ at C-4′, 1:0.9), 2.61 and 2.60 (s, H-2, 1:0.9),1.94 and 1.85 (s, H-5′’, 1:0.9)

(*R*)-MTPA ester of **4**: ^1^H NMR (Pyridin-d*_5_*): δ*_H_* 6.33 and 6.26 (s, H-6), 5.44 (t, H-3, overlapped), 2.95, 3.08, 3.19 and 3.21 (dd, H-4a, H-4b), 2.57 (s, H-12, overlapped), 1.36 and 1.37 (s, H-14), 1.29 and 1.30 (s, H-13)

^1^H NMR of **4** (Pyridin-*d_5_*): δ*_H_* 6.28 (s, H-6), 4.05 (t, H-3), 3.74 (s, OCH_3_ at C-7), 3.75 (s, OCH_3_ at C-5), 2.93 (dd, H-4a), 3.19 (dd, H-4b), 2.64 (s, H-12), 1.52 (s, H-13), 1.49 (s, H-14)

### 3.5. Cell Lines and Cell Culture

The human ovarian cancer cell line A2780 was obtained from European Collection of Cell Culture (ECACC, Salisbury, UK). A2780 cells were grown at 37 °C under humidified air supplemented with 5% CO_2_ in RPMI 1640 containing 10% heat inactivated fetal calf serum (Aidenbach, Germany, PAN Biotech), 120 IU/mL penicillin (PAN Biotech, Aidenbach, Germany), and 120 µg/mL streptomycin (PAN Biotech, Aidenbach, Germany). The cells were grown at 80% confluency before being used in further assays. The cultures of the cell line used are routinely tested for mycoplasma contamination. Results of STR analysis of A2780 can be found in Table S1.

### 3.6. Cytotoxicity Assay (Nuclear Shrinkage)

The cytotoxic effects of the isolated compounds were analyzed fluorescent based via measuring the shrinkage of cell nuclei (and subsequently increased average fluorescent intensity per cell nucleus) by staining cells with Hoechst-33342 and results were visualized with Array Scan XTI high content screening (HCS) system (Thermo Scientific, Wesel, Germany). Briefly, A2780 cells were seeded in 96-well-plates (Corning, Kaiserslautern, Germany) at a density of 4.000 *c*/*w*. Cells were treated with 10 µM and 100 µM of the compounds for 72 h. Then, medium was removed and 50 µL of nuclei staining solution (1.78 µM Hoechst-33342 in PBS) was added. Cells were incubated for 30 min at 37 °C in a humidified incubator before imaging. As a positive control for this assay, we have decided on a 24 h incubation with 100 µM cisplatin based on our experience. In principle, this incubation time could also be extended to 72 h—with the same results—but due to the severe toxicity, the number of objects (cells) that can be evaluated would be significantly reduced. In order to achieve a high significance by evaluation of many cells, we use a 24 h incubation time for 100 µM cisplatin.

### 3.7. Caspase 3/7-Activation Assay

Compound-induced activation of caspases 3 and 7 was analyzed using the CellEvent Caspase-3/7 green detection reagent (Thermo Scientific, Wesel, Germany) according to the manufacturer’s instructions. Briefly, A2780 cells were seeded in 96-well-plates (Corning, Kaiserslautern, Germany) at a density of 4.000 *c*/*w*. Cells were treated with 10 µM and 100 µM of the compounds for 72 h. Then, medium was removed and 50 µL of CellEvent Caspase 3/7 green detection reagent (2 µM in PBS supplemented with 5% heat inactivated FBS) was added. Cells were incubated for 30 min at 37 °C in a humidified incubator before imaging by using the Thermo Fisher ArrayScan XTI high content screening (HCS) system with a 10× magnification (Thermo Scientific). The pan caspase inhibitor QVD was used in a concentration of 20 µM diluted in the appropriate medium and incubated 30 min prior to compound addition. Based on our previous experience [73] cisplatin shows a strong induction of caspase 3/7 activity after 24 h incubation at high doses (100 µM), which was used as positive control in this study.

### 3.8. MTT-Assay

The rate of cell-survival under the action of test substances was evaluated by an improved MTT assay as previously described [73,74,75]. To investigate the effect of compound **2** cells were seeded at a density of 8000 *c*/*w* and incubated for 72 h with different concentrations of compound **2**. Cell survival was determined by addition of MTT (Serva, Heidelberg, Germany) solution (5 mg/mL in phosphate buffered saline). The formazan precipitate was dissolved in DMSO (VWR, Langenfeld, Germany). Absorbance was measured at 544 nm and 690 nm in a FLUOstar microplate reader (BMG LabTech, Offenburg, Germany).

### 3.9. Data Analysis

Concentration-effect curves for calculation of IC_50_ values were constructed with Prism 7.0 (GraphPad, San Diego, CA, USA) by fitting the pooled data from at least three independent experiments performed in triplicates to the four-parameter logistic equation. Bar graphs were also constructed with Prism 7.0 (GraphPad, San Diego, CA, USA). The results of the assays were tested for normal distribution using the Shapiro-Wilk test and an online tool [76]. Normal distribution is given. Statistical analysis was performed using unpaired two-tailed t-test. To normalize the cytotoxic effects and the effects on caspase3/7-activation, fluorescence values for vehicle controls were set to 0% and values for 24 h 100 µM cisplatin were set to 100%.

## Figures and Tables

**Table 1:** ^1^H- and ^13^C-NMR data of **1** and **2** (600 and 150 MHz, δ in ppm, in DMSO-*d*_6_).

No.	1			2		
	δ_C_	Type	δ_H_ (*J* in Hz)	δ_C_	Type	δ_H_ (*J* in Hz)
1	203.0	C	-	203.1	C	
2	32.9	CH_3_	2.56 s	32.9	CH_3_	2.57 s
1′	105.0	C	-	104.7	C	-
2′	163.1	C	-	162.3	C	-
3′	106.0	C	-	108.5	C	-
4′	164.1	C	-	163.3	C	-
5′	87.1	CH	6.21 s	87.3	CH	6.23 s
6′	161.8	C	-	161.8	C	-
1″	28.6	CH_2_	2.62 dd (13.0/6.6)	20.8	CH_2_	3.13 d (7.2)
			2.71 dd (13.0/7.7)			
2″	73.5	CH	4.14 m	122.6	CH	5.07 t (7.2/1)
3″	148.1	C	-	130.3	C	-
4″	109.7	CH_2_	4.51 m	25.5	CH_3_	1.59 s
			4.54 m			
5″	16.9	CH_3_	1.69 s	17.6	CH_3_	1.68 s
OCH_3_ at C-4′	55.8	CH_3_	3.87 s	56.0	CH_3_	3.90 s
OCH_3_ at C-6′	55.9	CH_3_	3.92 s	55.9	CH_3_	3.92 s
OH at C-2′	-		13.98 s	-	-	13.95 s
OH at C-2″	-		4.63 d	-		-

**Table 2:** ^1^H-and ^13^C-NMR data of **3** and **4** (600 and 150 MHz, δ in ppm, in CDCl_3_).

No.		3			4	
	δ_C_	Type	δ_H_ (*J* in Hz)	δ_C_	Type	δ_H_ (*J* in Hz)
2	80.9	C	-	77.7	C	-
3	123.0	CH	5.38 d (10.1)	69.3	CH	3.77 t (5.2/5.5)
4a	116.8	CH	6.59 d (10.1)	26.2	CH	2.62 dd (17.1/5.5)
4b						2.84 dd (17.1/5.2)
5	161.2	C	-	159.7	C	-
6	91.9	CH	5.99 s	88.1	CH	6.07 s
7	166.7	C	-	156.8	C	-
8	106.0	C	-	113.8	C	-
9	156.7	C	-	151.5	C	-
10	102.7	C	-	100.9	C	-
11	202.9	C	-	201.6	C	-
12	33.1	CH_3_	2.66 s	32.7	CH_3_	2.47 s
13 ^a^	26.7	CH_3_	1.43 s	22.0	CH_3_	1.33 s
14 ^a^	41.7	CH_2_	1.79 m	24.8	CH_3_	1.31 s
1′	23.0	CH_2_	2.10 m	-	-	-
2′	123.6	CH	5.09 t (7.1/1.5)	-	-	-
3′	132.9	C	-	-	-	-
4′ ^a^	25.9	CH_3_	1.57 s	-	-	-
5′ ^a^	17.3	CH_3_	1.66 s	-	-	-
OCH_3_ at C-5	55.7	CH_3_	3.83 s	55.7	CH_3_	3.84 s
OCH_3_ at C-7	-	-	-	56.2	CH_3_	3.80 s
OH at C-7	-	-	13.84 s	-	-	-

^a^ assignments interchangeable (compound **3** only).

**Table 3:** IC_50_ and pIC_50_ for compound 2 and cisplatin in nuclear shrinkage and MTT assay.

	Nuclear Shrinkage Assay		MTT Assay	
	IC_50_ [µM]	pIC_50_ ± SEM	IC_50_ [µM]	pIC_50_ ± SEM
**Compound 2**	30.0	4.52 ± 0.11	>100	<4
**Cisplatin**	4.65	5.33 ± 0.07	6.42	5.19 ± 0.01

Data shown are corresponding to Figure 6 and are the mean of pooled data from at least three experiments.

## Supplementary Materials

The following are available online. Figure S1–S31: HRMS, 1D and 2D NMR spectra of **1**–**7**; Figure S32: Cytotoxic activity of compounds of *Melicope barbigera*; Figure S33: Effects on caspase 3/7-activation of cytotoxic compounds of *Melicope barbigera*; Table S1: Results of STR analysis of A2780.
