# Supplementary material for: New Acetophenones and Chromenes from the Leaves of Melicope barbigera A. Gray
Source: Molecules. 2021 Jan 28;26(3):688. doi: 10.3390/molecules26030688 (PMC7865373; doi:10.3390/molecules26030688)
Supplement: Supplementary file 1 [file molecules-26-00688-s001.pdf]

Supplementary Material

New acetophenones and chromenes from the leaves of *Melicope barbigera* A. Gray

Table of content

Figure S1. HRESIMS of compound **1.** 4

Figure S2. ^{1}H NMR (600 MHz, DMSO-*d*$_6$*)* spectrum of compound **1.** 5

Figure S3.^{13}C NMR (600 MHz, DMSO-d$_6$) spectrum of compound **1.** 6

Figure S4. COSY (600 MHz, DMSO-*d*$_6$) spectrum of compound **1**. 7

Figure S5. HSQC (600 MHz/150 MHz, DMSO-*d*$_6$) spectrum of compound **1**. 8

Figure S6. HMBC (600 MHz/150 MHz, DMSO-*d*$_6$) spectrum of compound **1**. 9

Figure S7. HRESIMS of compound **2**. 10

Figure S8. ^{1}H NMR (600 MHz, DMSO-*d*$_6$) spectrum of compound **2.** 11

Figure S9. ^{13}C NMR (150 MHz, DMSO-*d*$_6$) spectrum of compound **2.** 12

Figure S10. COSY (600 MHz, DMSO-*d*$_6$) spectrum of compound **2**. 13

Figure S11. HSQC (600 MHz/150 MHz, DMSO-*d*$_6$) spectrum of compound **2**. 14

Figure S12. HMBC (600 MHz/150 MHz, DMSO-*d*$_6$) spectrum of compound **2**. 15

Figure S13. HRESIMS of compound **3**. 16

Figure S14. ^{1}H NMR (600 MHz, $CDCl_3$) spectrum of compound **3**. 17

Figure S15. ^{13}C NMR (150 MHz, $CDCl_3$) spectrum of compound **3.** 18

Figure S16. COSY (600 MHz, $CDCl_3$) spectrum of compound **3.** 19

Figure S17. HSQC (600 MHz/150 MHz, $CDCl_3$) spectrum of compound **3** 20

Figure S18. HMBC (600 MHz/150MHz, $CDCl_3$) spectrum of compound **3.** 21

Figure S19. HRESIMS of compound **4**. 22

Figure S20. ^{1}H NMR (600 MHz, $CDCl_3$) spectrum of compound **4**. 23

Figure S21. ^{13}C NMR (125 MHz, $CDCl_3$) spectrum of compound **4.** 24

Figure S22. COSY (600 MHz, $CDCl_3$) spectrum of compound **4.** 25

Figure S23. HSQC (600 MHz/150 MHz, $CDCl_3$) spectrum of compound **4.** 26

Figure S24. HMBC (600 MHz/150 MHz, $CDCl_3$) spectrum of compound **4** 27

Figure S25. ROESY (600 MHz, $CDCl_3$) spectrum of compound **4** 28

Figure S26. ^{1}H NMR (600 MHz, pyridine-*d*$_5$) spectrum of compound **4** 29

Figure S27. ^{1}HNMR spectra of **4** after reaction with (*S*)-MTPA reagent in pyridine-*d*$_5$ 30

Figure S28. HRESIMS of compound **5**. 31

Figure S29. ^{1}H NMR (600 MHz, $CDCl_3$) spectrum of compound **5**. 31

Figure S30. HRESIMS of compound **6+7**. 32
Figure S31.^{1}H NMR (600 MHz, $CDCl_3$) spectrum of compound **6+7**. 32
Figure S32. Cytotoxic activity of compounds of *Melicope barbigera* 35
Figure S33. Effects on caspase 3/7-activation of cytotoxic compounds of *Melicope barbigera*........ 36
Table S1. Results of STR analysis of A2780..................... 36

Acquisition Parameter

Source Type	ESI	Ion Polarity	Positive	Set Nebulizer	0.3 Bar
Focus	Not active	Set Capillary	4000 V	Set Dry Heater	180 °C
Scan Begin	50 m/z	Set End Plate Offset	-500 V	Set Dry Gas	4.0 l/min
Scan End	1500 m/z	Set Collision Cell RF	600.0 Vpp	Set Divert Valve	Source

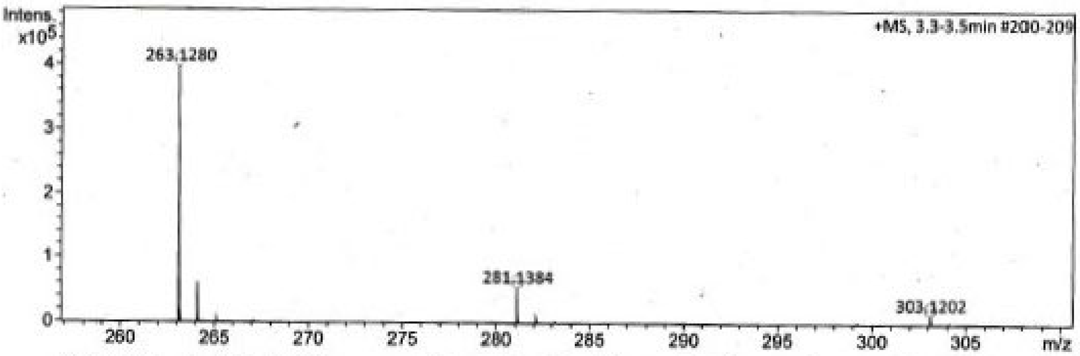

Meas. m/z	#	Ion Formula	m/z	err [ppm]	mSigma	# mSigma	Score	rdb	e⁻ Conf	N-Rule
263.1280	1	C15H19O4	263.1278	-0.7	7.4	1	100.00	6.5	even	ok
	2	C16H15N4	263.1291	4.4	20.8	2	46.76	11.5	even	ok
281.1384	1	C15H21O5	281.1384	-0.0	2.1	1	100.00	5.5	even	ok
	2	C16H17N4O	281.1397	4.7	15.7	2	39.68	10.5	even	ok
303.1202	1	C15H20NaO5	303.1203	0.4	4.1	1	100.00	5.5	even	ok

Figure S1. HRESIMS of compound **1**.

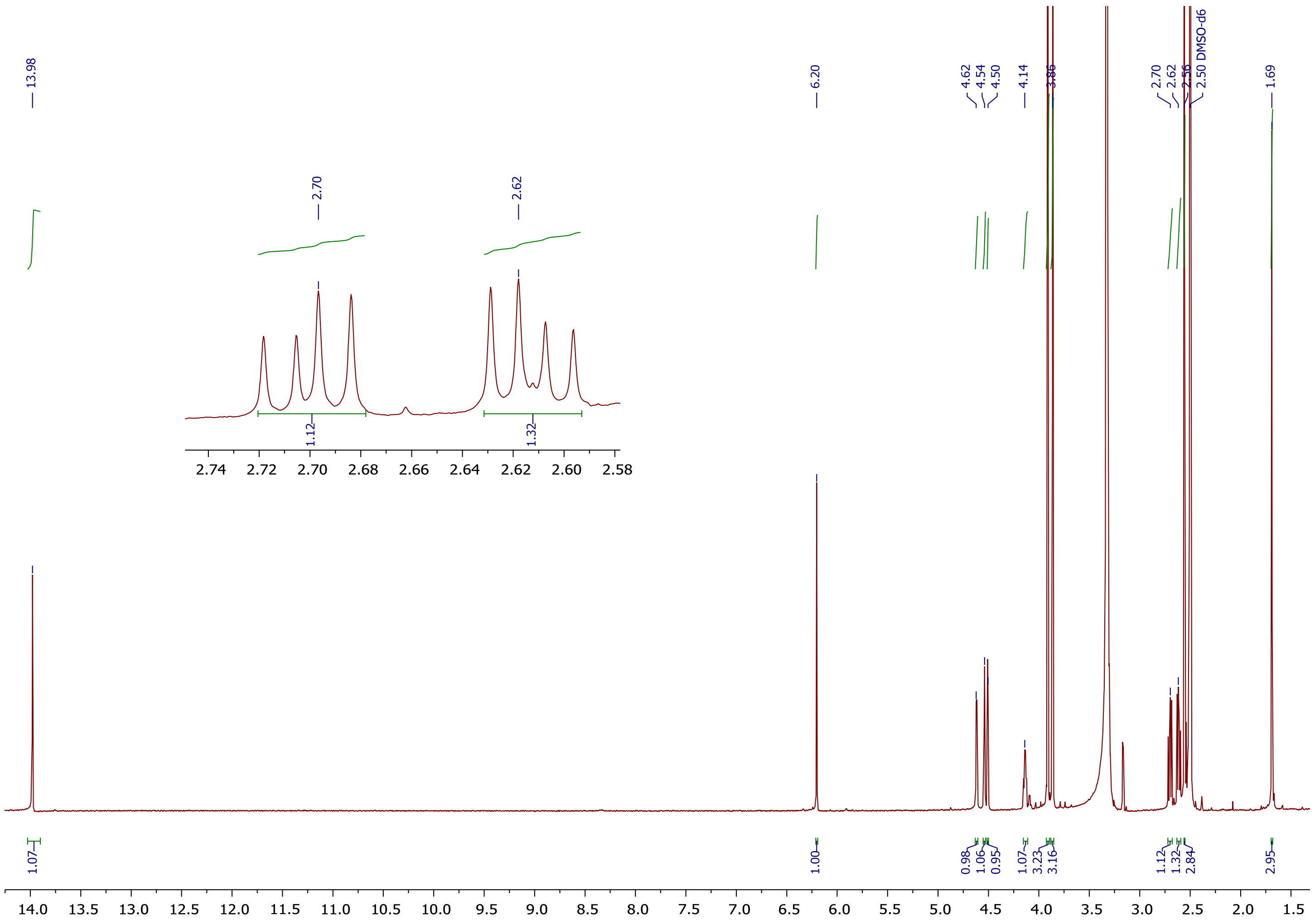

Figure S2. ^{1}H NMR (600 MHz, DMSO-d_6) spectrum of compound **1**.

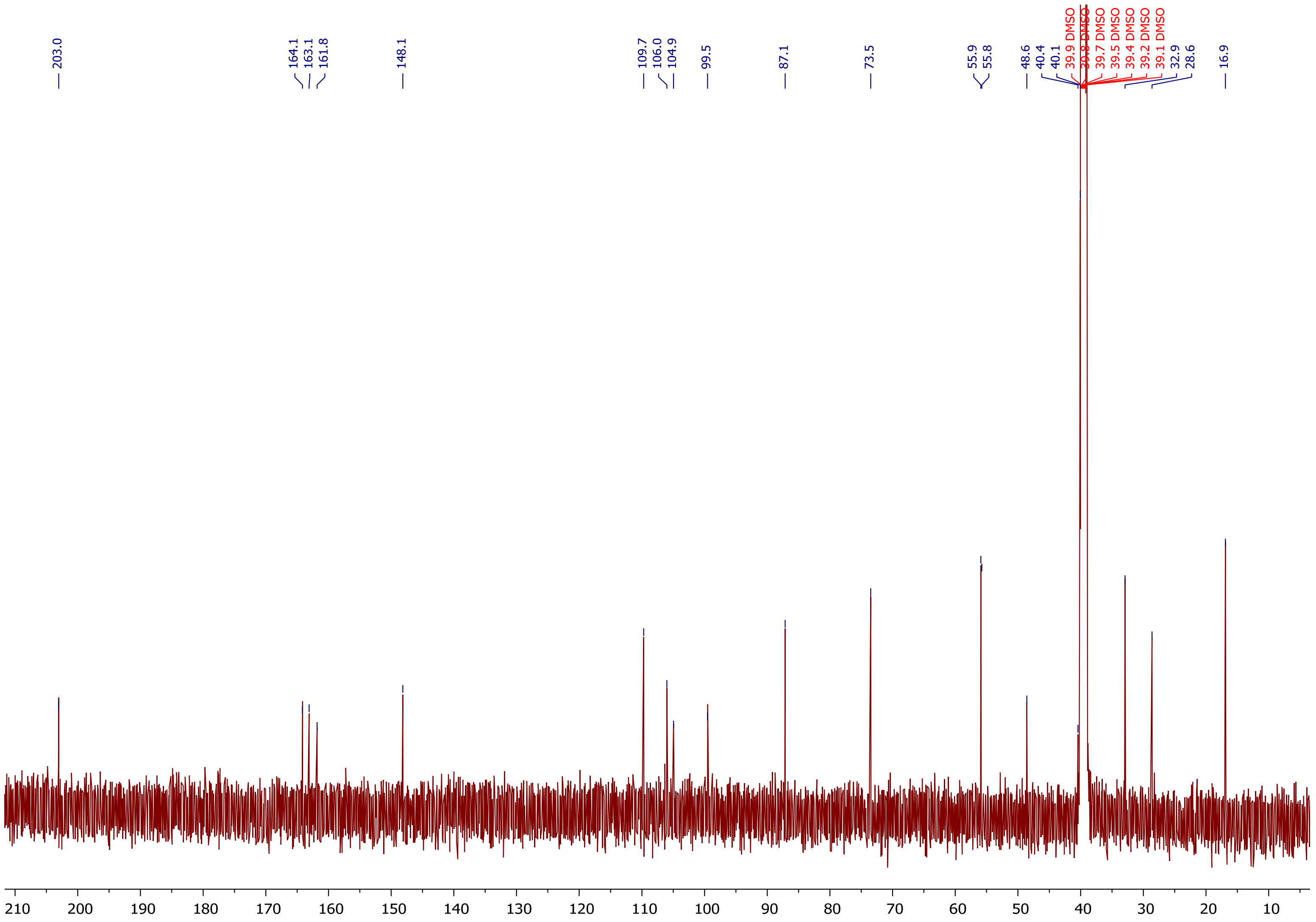

Figure S3. ^{13}C NMR (600 MHz, DMSO-d_6) spectrum of compound **1**.

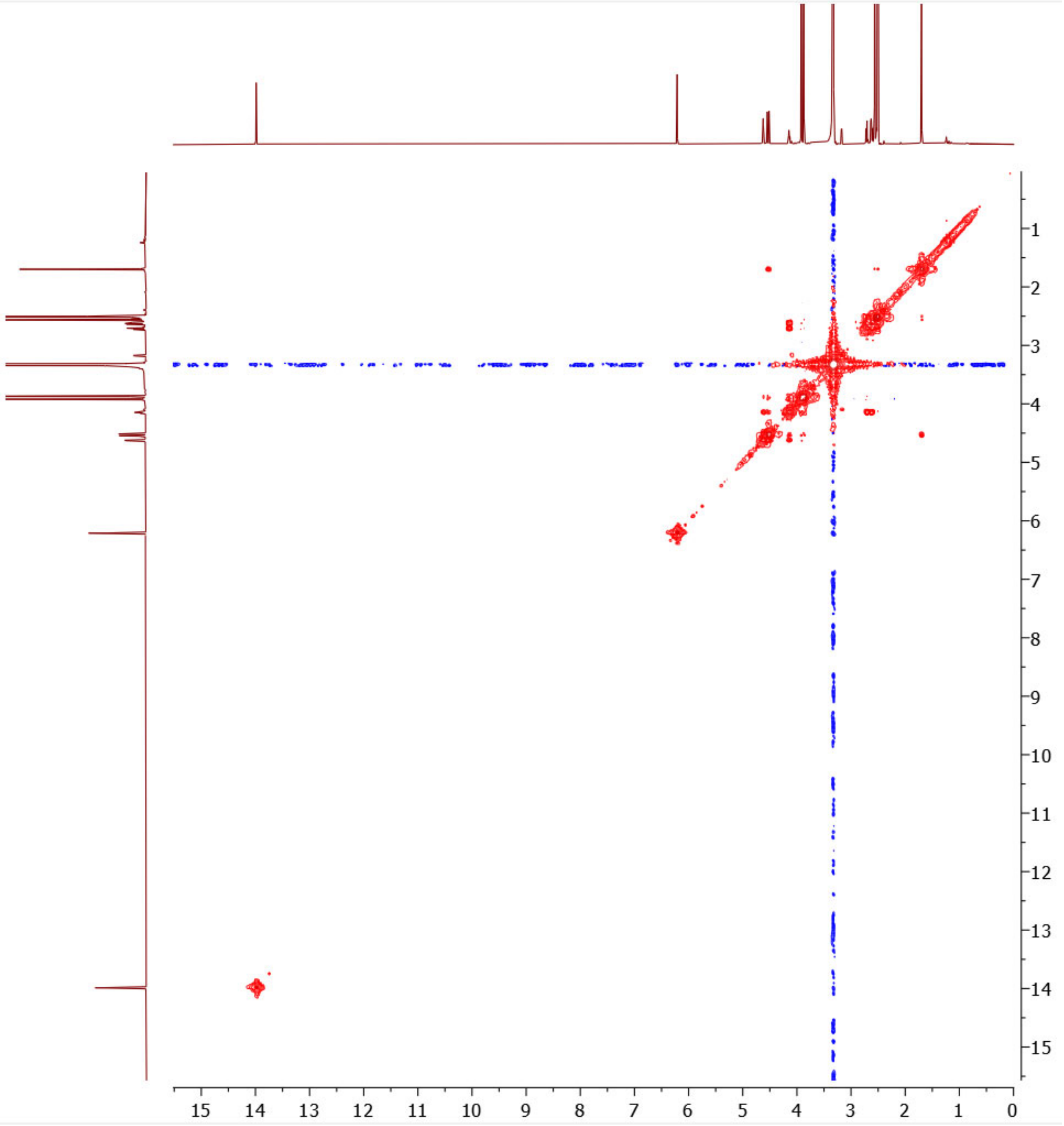

Figure S4. COSY (600 MHz, DMSO-d_6) spectrum of compound **1**.

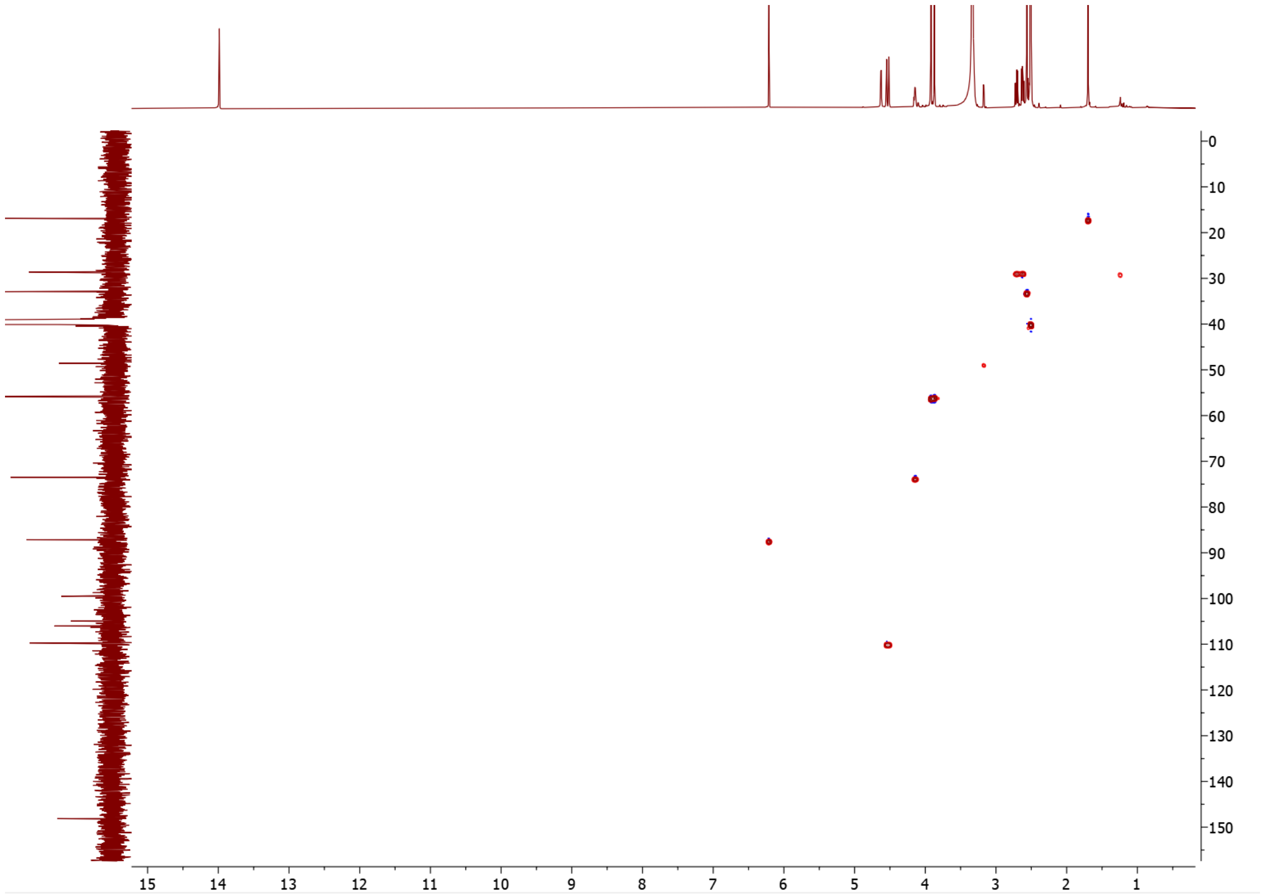

Figure S5. HSQC (600 MHz/150 MHz, DMSO-d_6) spectrum of compound **1**.

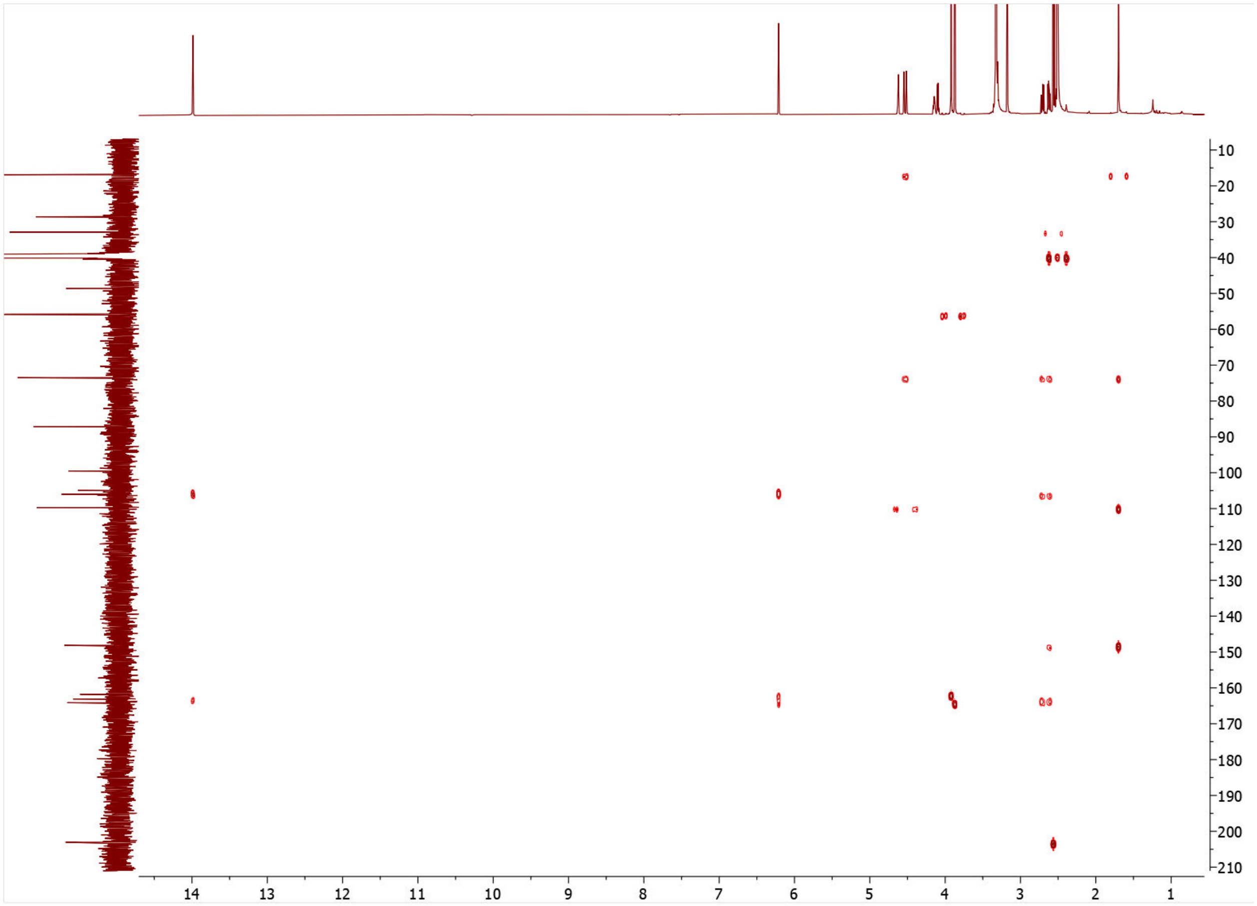

Figure S6. HMBC (600 MHz/150 MHz, DMSO-d_6) spectrum of compound **1**.

Acquisition Parameter

Source Type	ESI	Ion Polarity	Positive	Set Nebulizer	0.3 Bar
Focus	Not active	Set Capillary	4000 V	Set Dry Heater	180 °C
Scan Begin	50 m/z	Set End Plate Offset	-500 V	Set Dry Gas	4.0 l/min
Scan End	1500 m/z	Set Collision Cell RF	600.0 Vpp	Set Divert Valve	Source

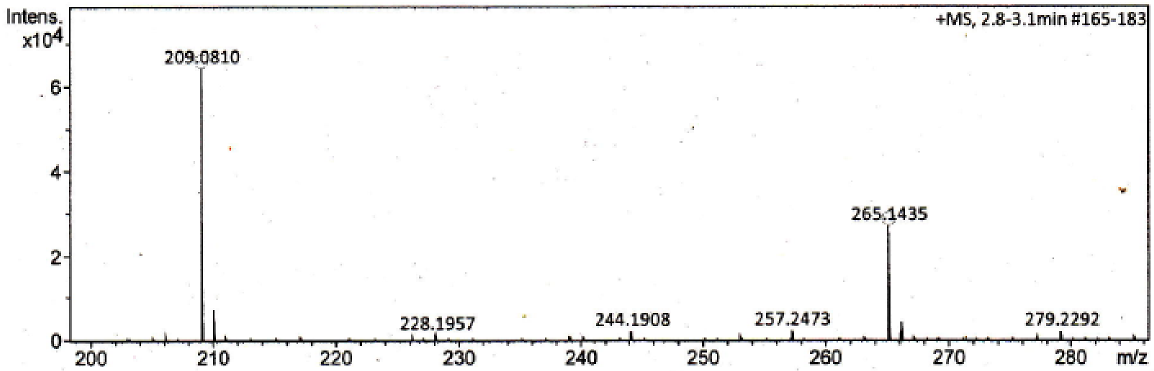

Meas. m/z	#	Ion Formula	m/z	err [ppm]	mSigma	# mSigma	Score	rdb	e⁻ Conf	N-Rule
209.0810	1	$C_{11}H_{13}O_4$	209.0808	-0.7	3.4	1	100.00	5.5	even	ok
265.1435	1	$C_{15}H_{21}O_4$	265.1434	-0.2	5.3	1	100.00	5.5	even	ok

Figure S7. HRESIMS of compound **2**.

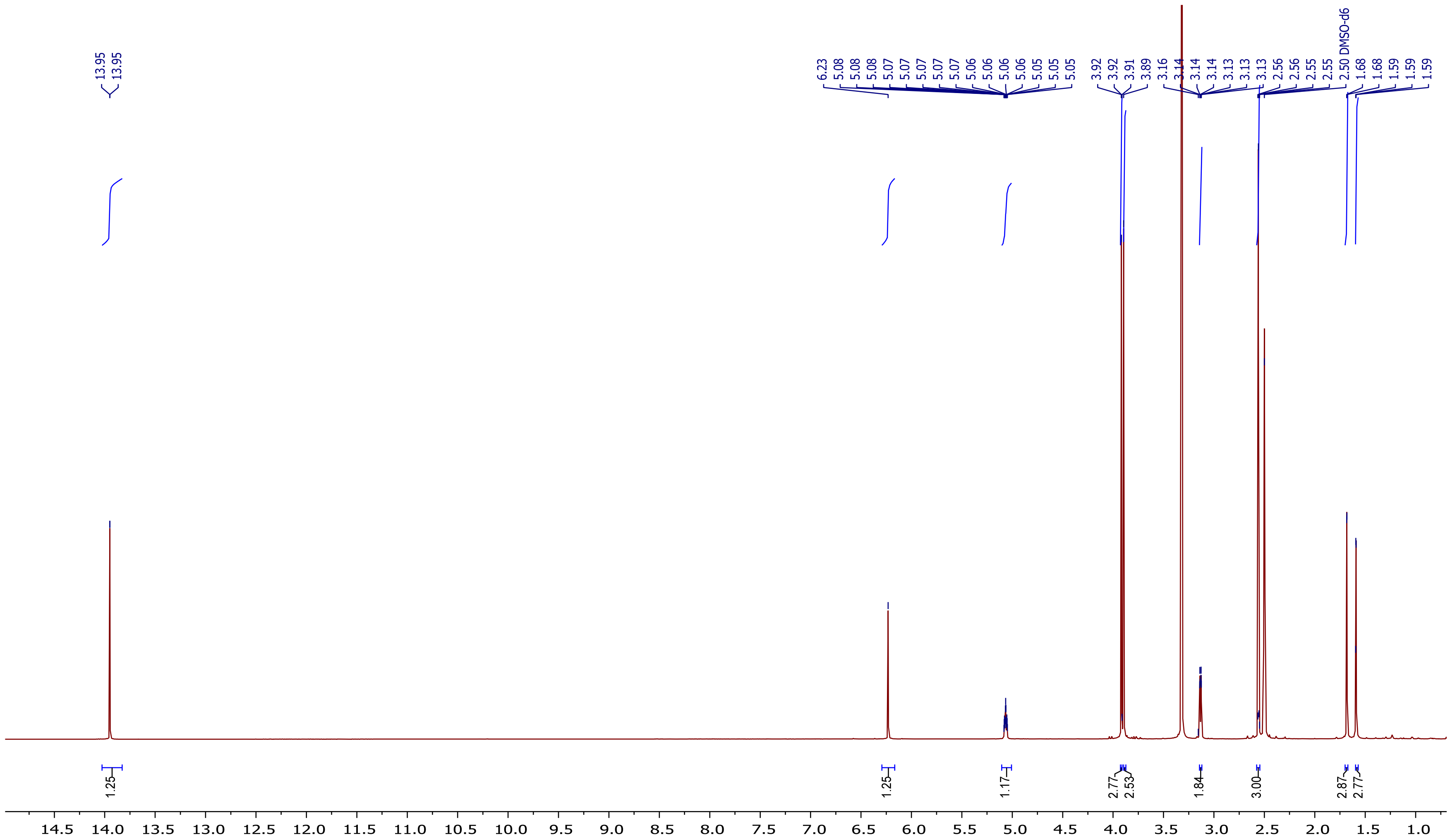

Figure S8. ^{1}H NMR (600 MHz, DMSO-d_6) spectrum of compound **2**.

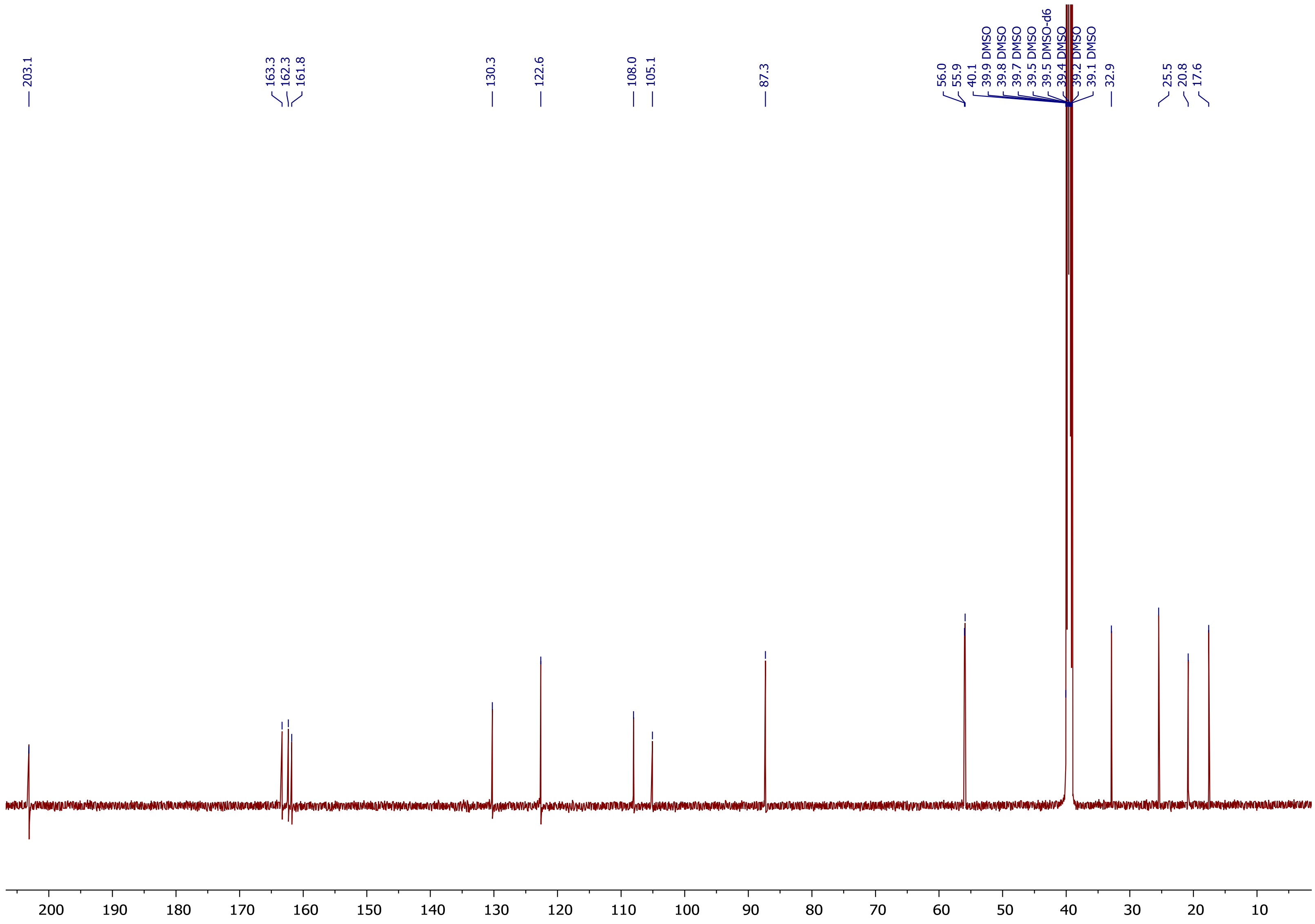

Figure S9. ^{13}C NMR (150 MHz, DMSO-d_6) spectrum of compound **2**.

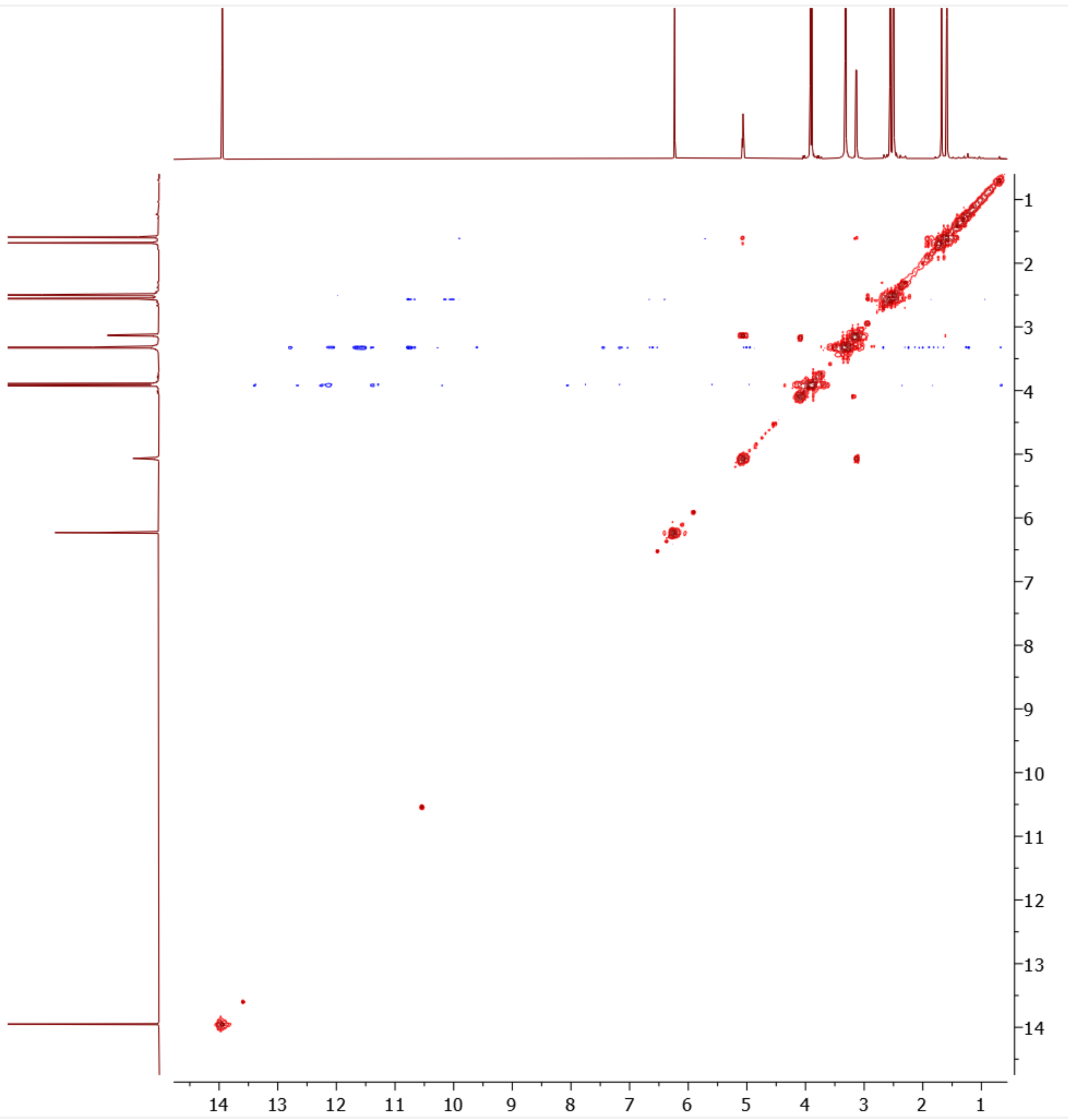

Figure S10. COSY (600 MHz, DMSO-d_6) spectrum of compound **2**.

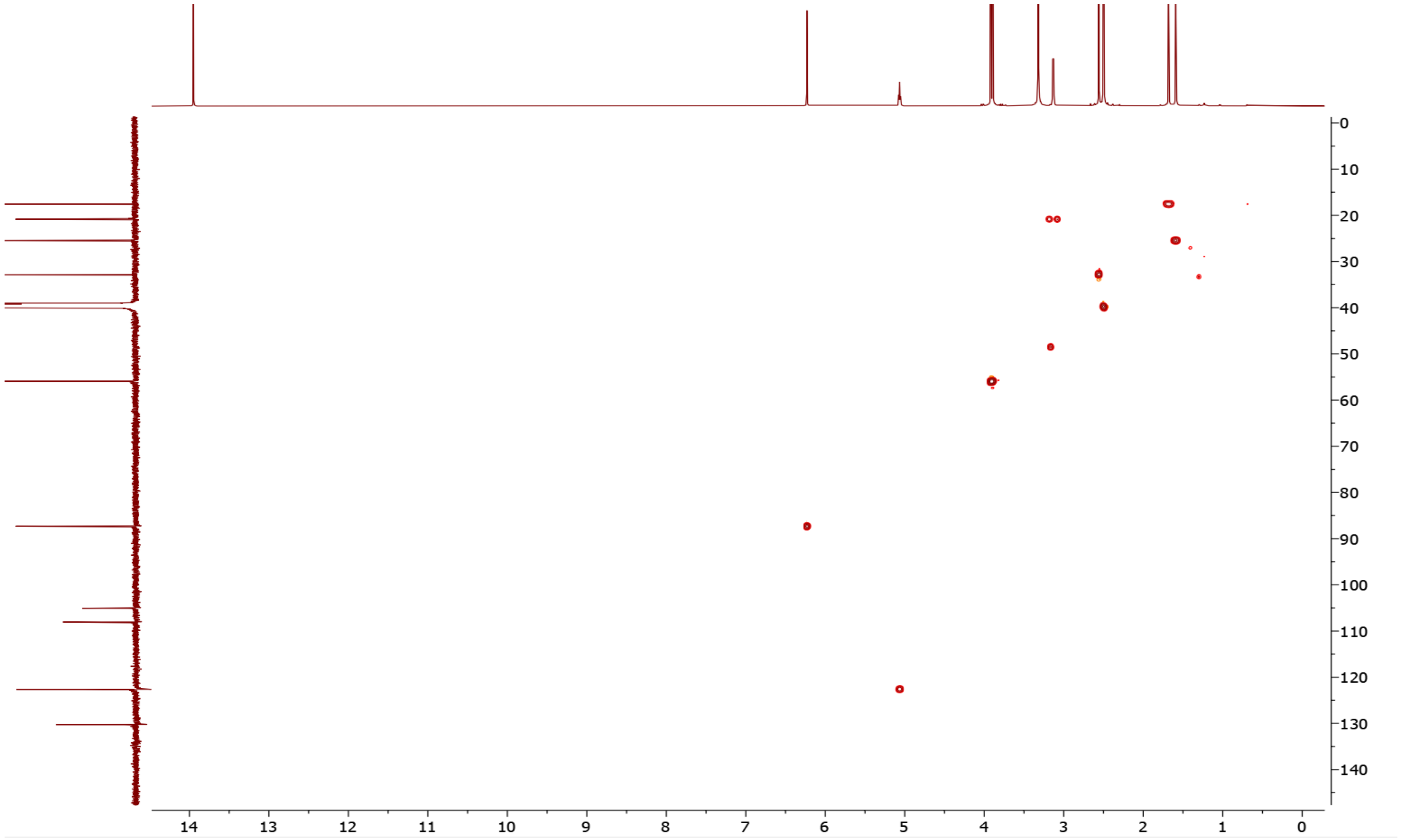

Figure S11. HSQC (600 MHz/150 MHz, DMSO-d_6) spectrum of compound **2**.

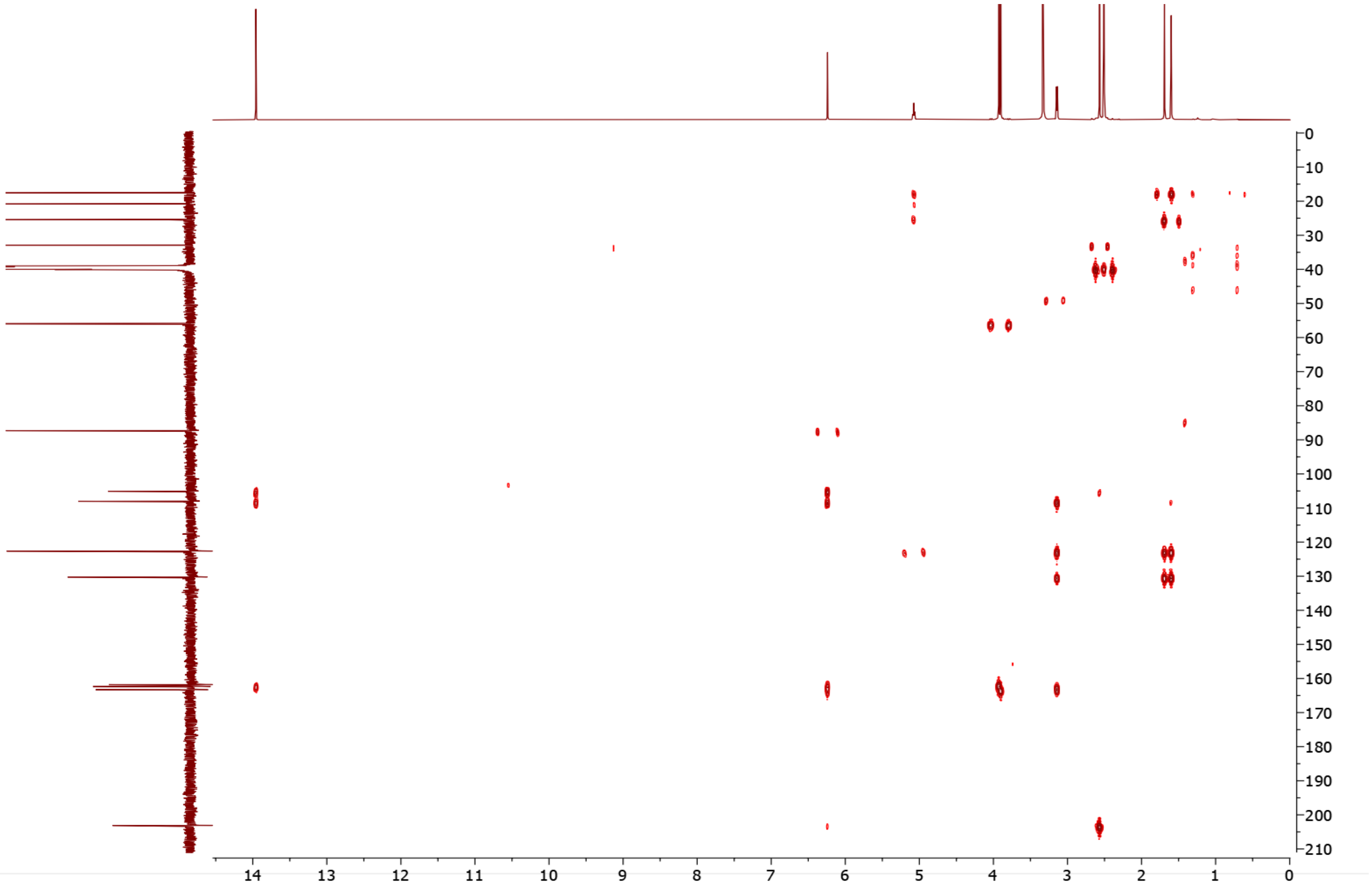

Figure S12. HMBC (600 MHz/150 MHz, DMSO-d_6) spectrum of compound **2**.

Acquisition Parameter

Source Type	ESI	Ion Polarity	Positive	Set Nebulizer	0.3 Bar
Focus	Not active	Set Capillary	4000 V	Set Dry Heater	180 °C
Scan Begin	50 m/z	Set End Plate Offset	-500 V	Set Dry Gas	4.0 l/min
Scan End	1500 m/z	Set Collision Cell RF	600.0 Vpp	Set Divert Valve	Source

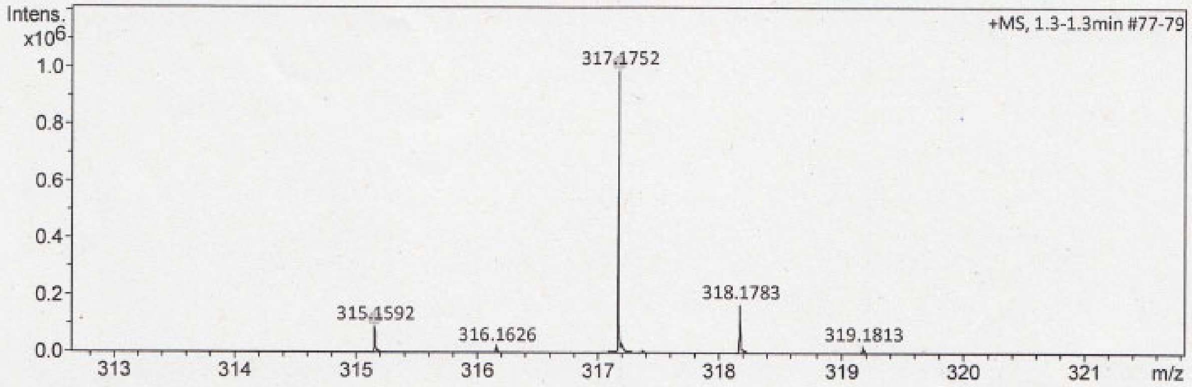

Meas. m/z	#	Ion Formula	m/z	err [ppm]	mSigma	# mSigma	Score	rdb	e⁻ Conf	N-Rule
315.1592	1	C19H23O4	315.1591	-0.2	775.1	1	100.00	8.5	even	ok
317.1752	1	C19H25O4	317.1747	-1.3	21.6	1	100.00	7.5	even	ok
	2	C20H21N4	317.1761	2.9	34.9	2	56.91	12.5	even	ok

Figure S13. HRESIMS of compound **3**.

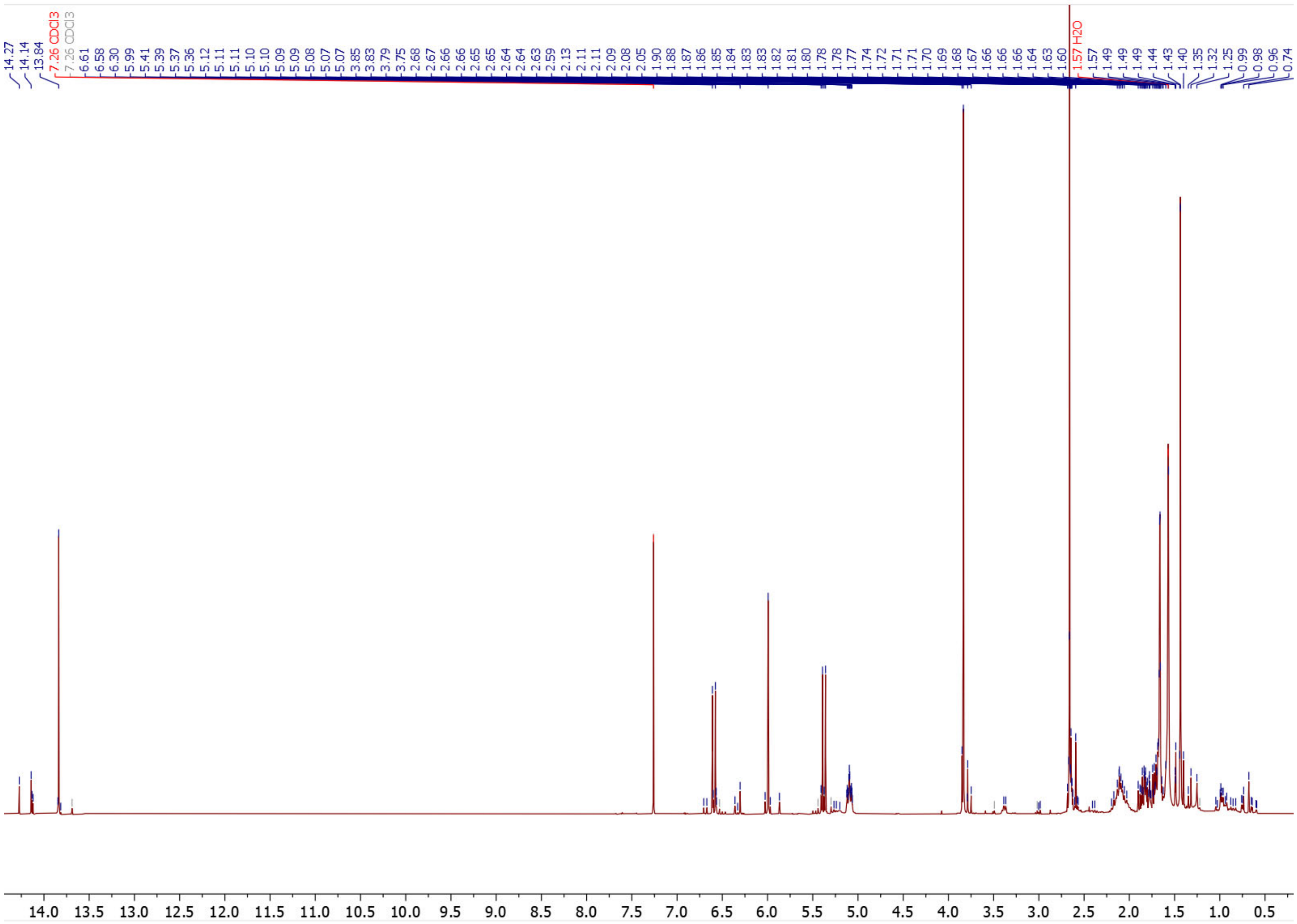

Figure S14. ^{1}H NMR (600 MHz, $CDCl_3$) spectrum of compound **3**.

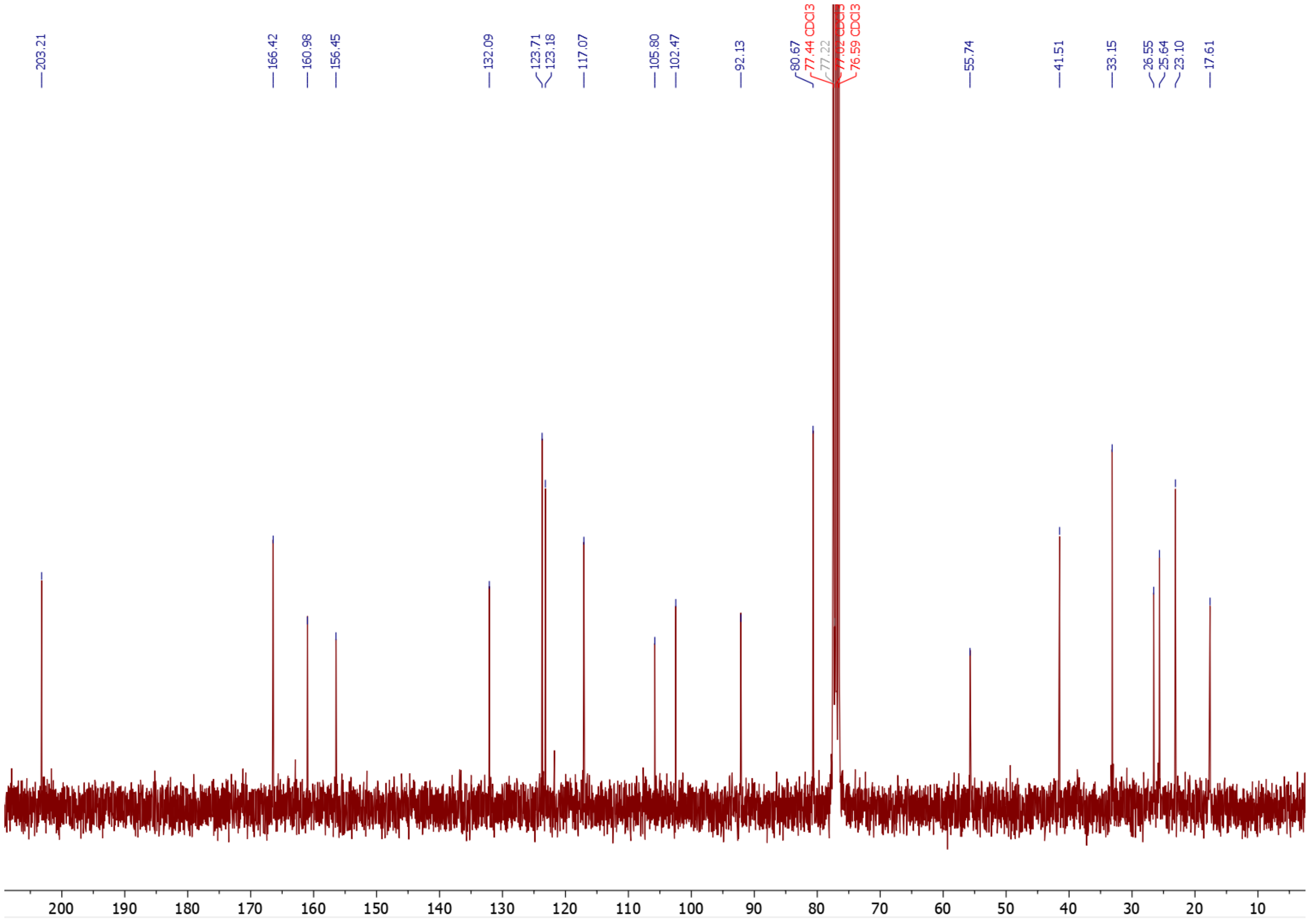

Figure S15. ^{13}C NMR (150 MHz, $CDCl_3$) spectrum of compound **3**.

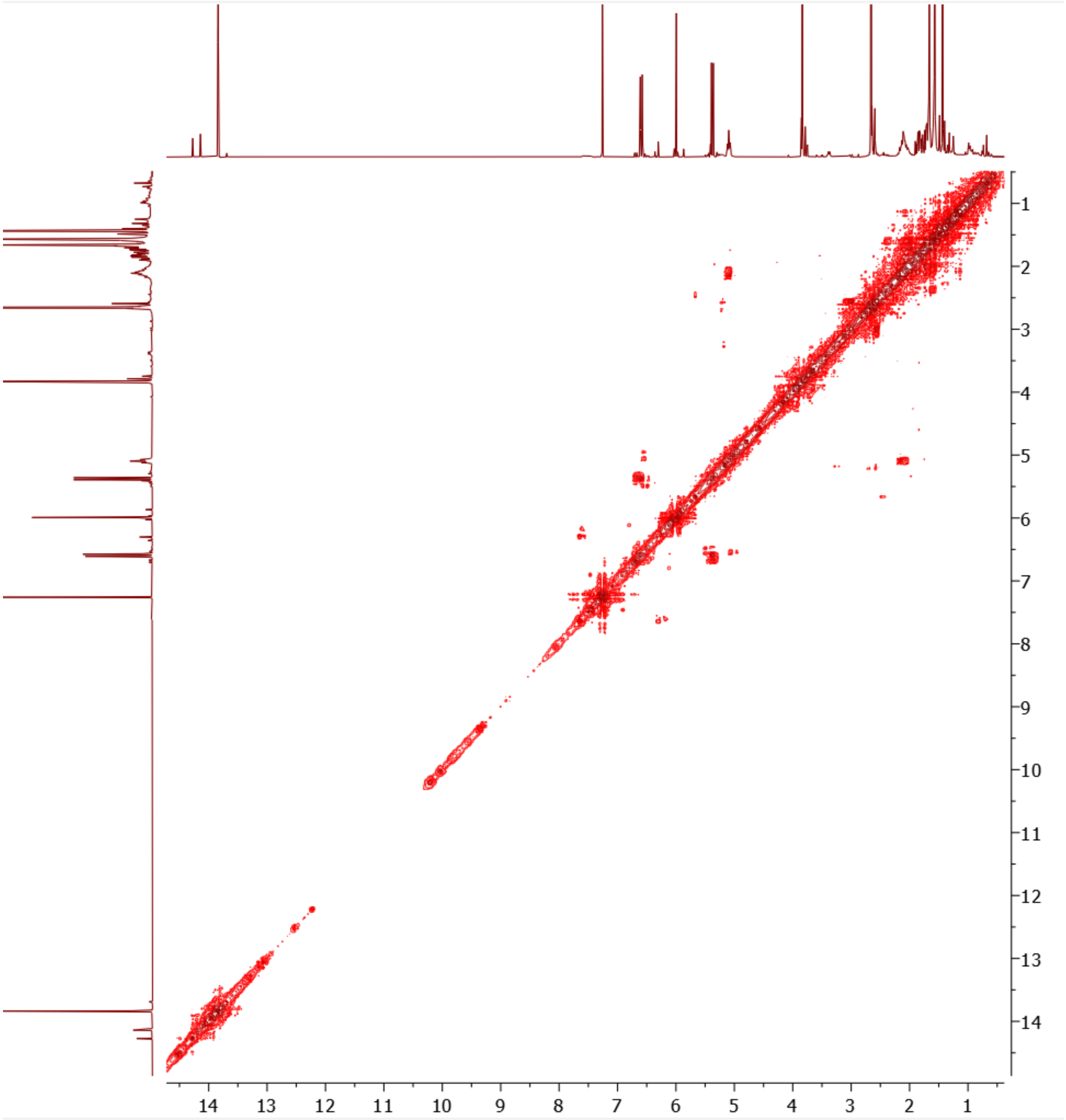

Figure S16. COSY (600 MHz, $CDCl_3$) spectrum of compound **3**.

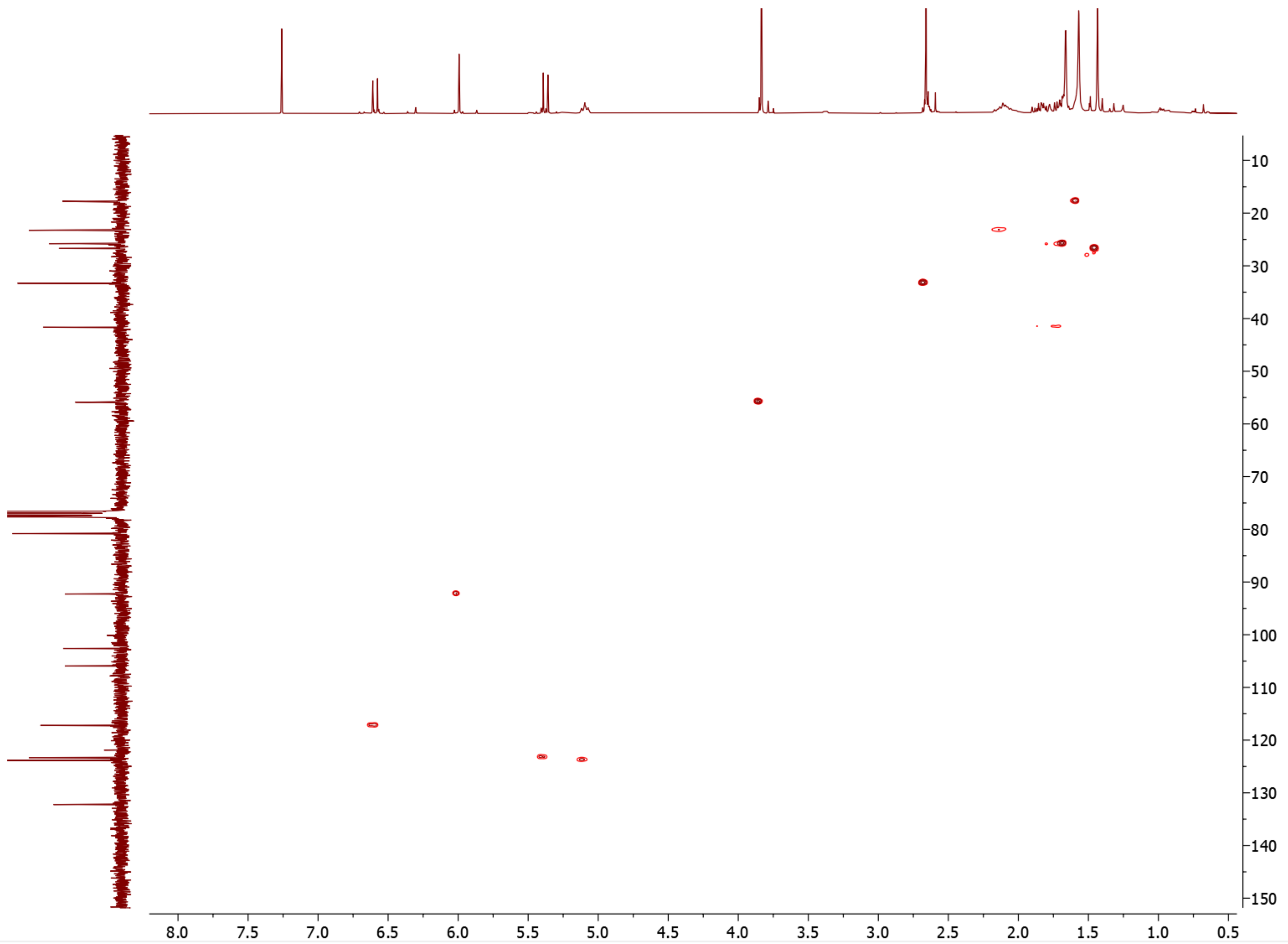

Figure S17. HSQC (600 MHz/150 MHz, $CDCl_3$) spectrum of compound **3**

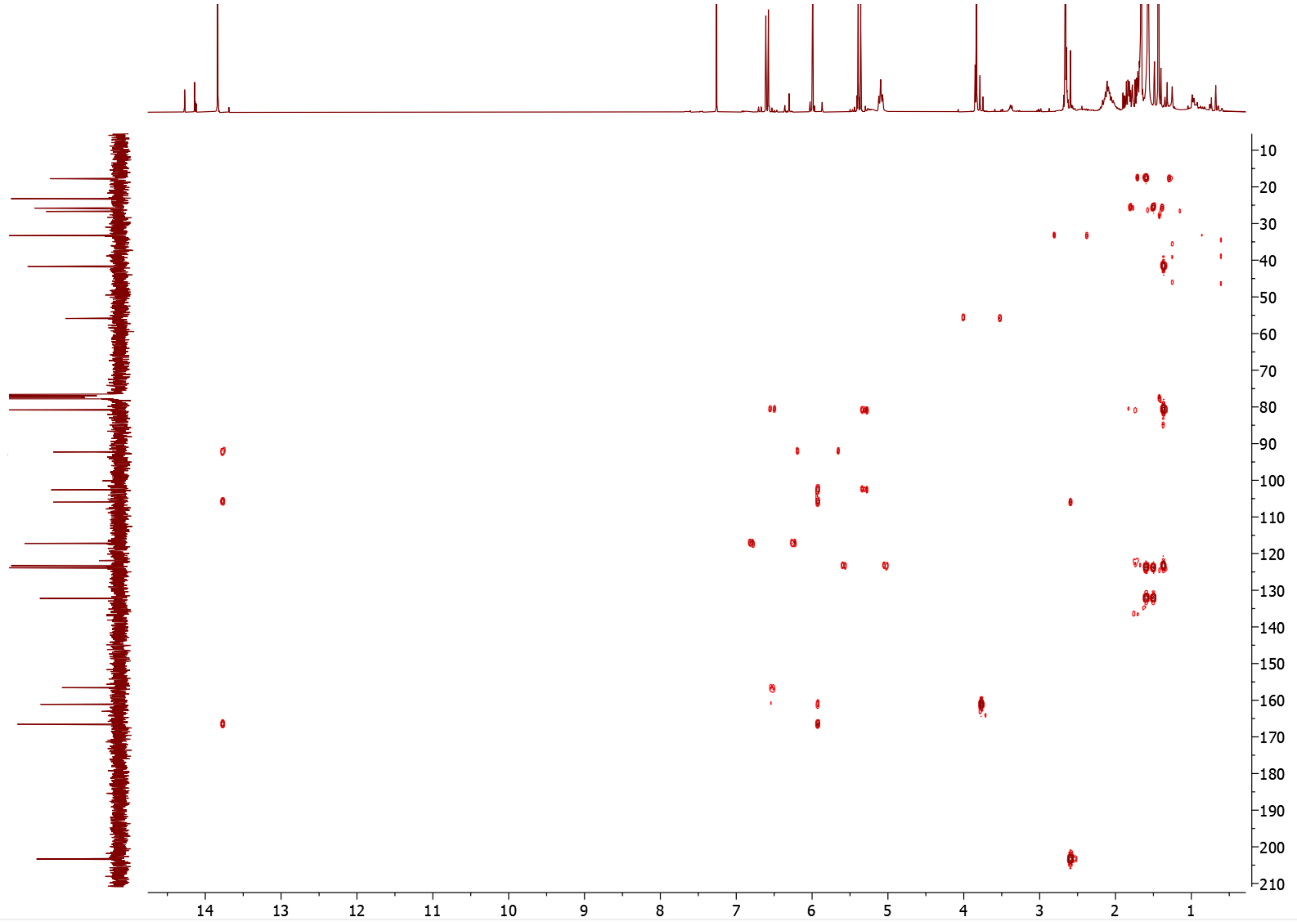

Figure S18. HMBC (600 MHz/150MHz, $CDCl_3$) spectrum of compound **3**.

Acquisition Parameter

Source Type	ESI	Ion Polarity	Positive	Set Nebulizer	0.3 Bar
Focus	Not active	Set Capillary	4000 V	Set Dry Heater	180 °C
Scan Begin	50 m/z	Set End Plate Offset	-500 V	Set Dry Gas	4.0 l/min
Scan End	1500 m/z	Set Collision Cell RF	600.0 Vpp	Set Divert Valve	Source

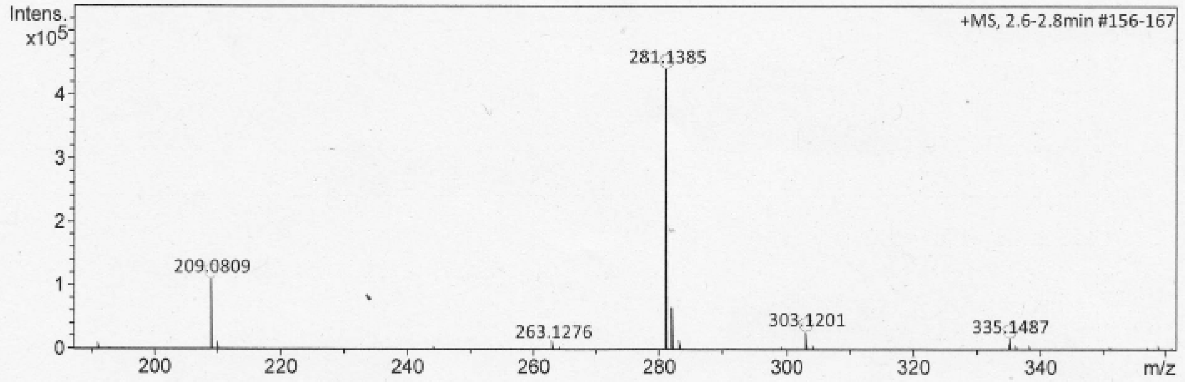

Meas. m/z	#	Ion Formula	m/z	err [ppm]	mSigma	# mSigma	Score	rdb	e⁻ Conf	N-Rule
209.0809	1	C11H13O4	209.0808	-0.4	3.3	1	100.00	5.5	even	ok
281.1385	1	C15H21O5	281.1384	-0.5	10.5	1	100.00	5.5	even	ok
303.1201	1	C15H20NaO5	303.1203	0.8	6.2	1	100.00	5.5	even	ok
335.1487	1	C18H23O6	335.1489	0.7	7.4	1	100.00	7.5	even	ok

Figure S19. HRESIMS of compound **4**.

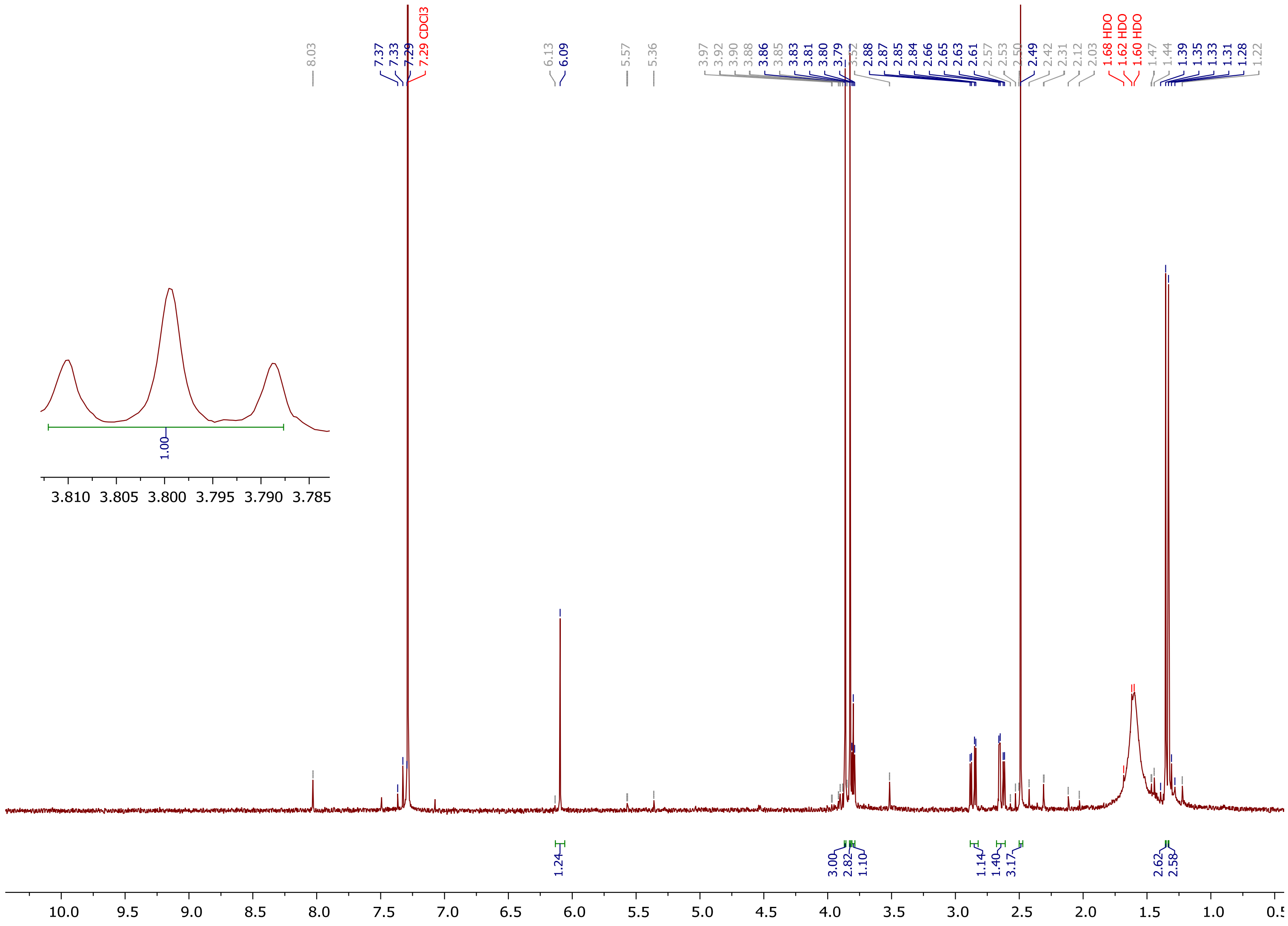

Figure S20. ^{1}H NMR (600 MHz, $CDCl_3$) spectrum of compound **4**.

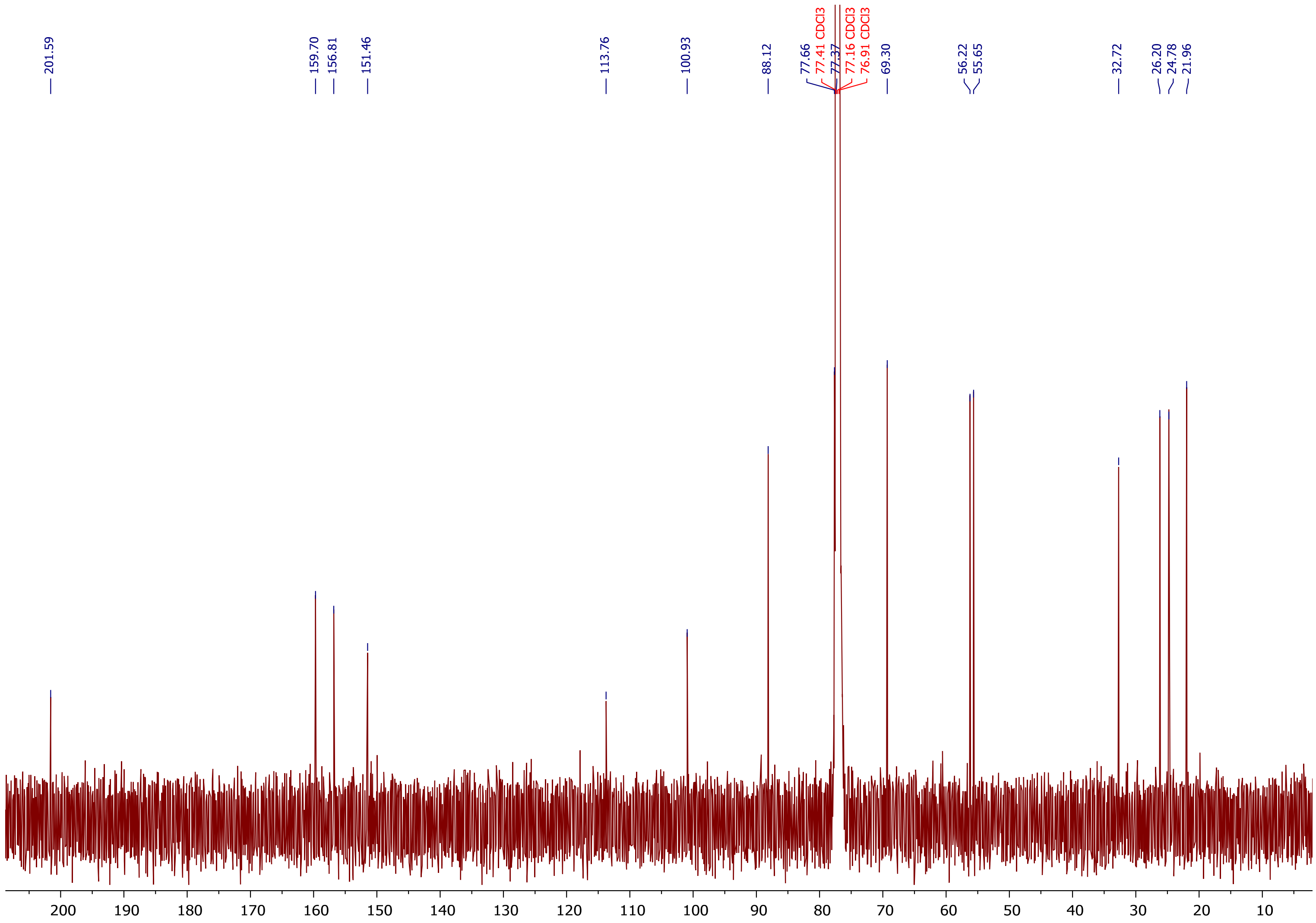

Figure S21. ^{13}C NMR (125 MHz, $CDCl_3$) spectrum of compound **4**.

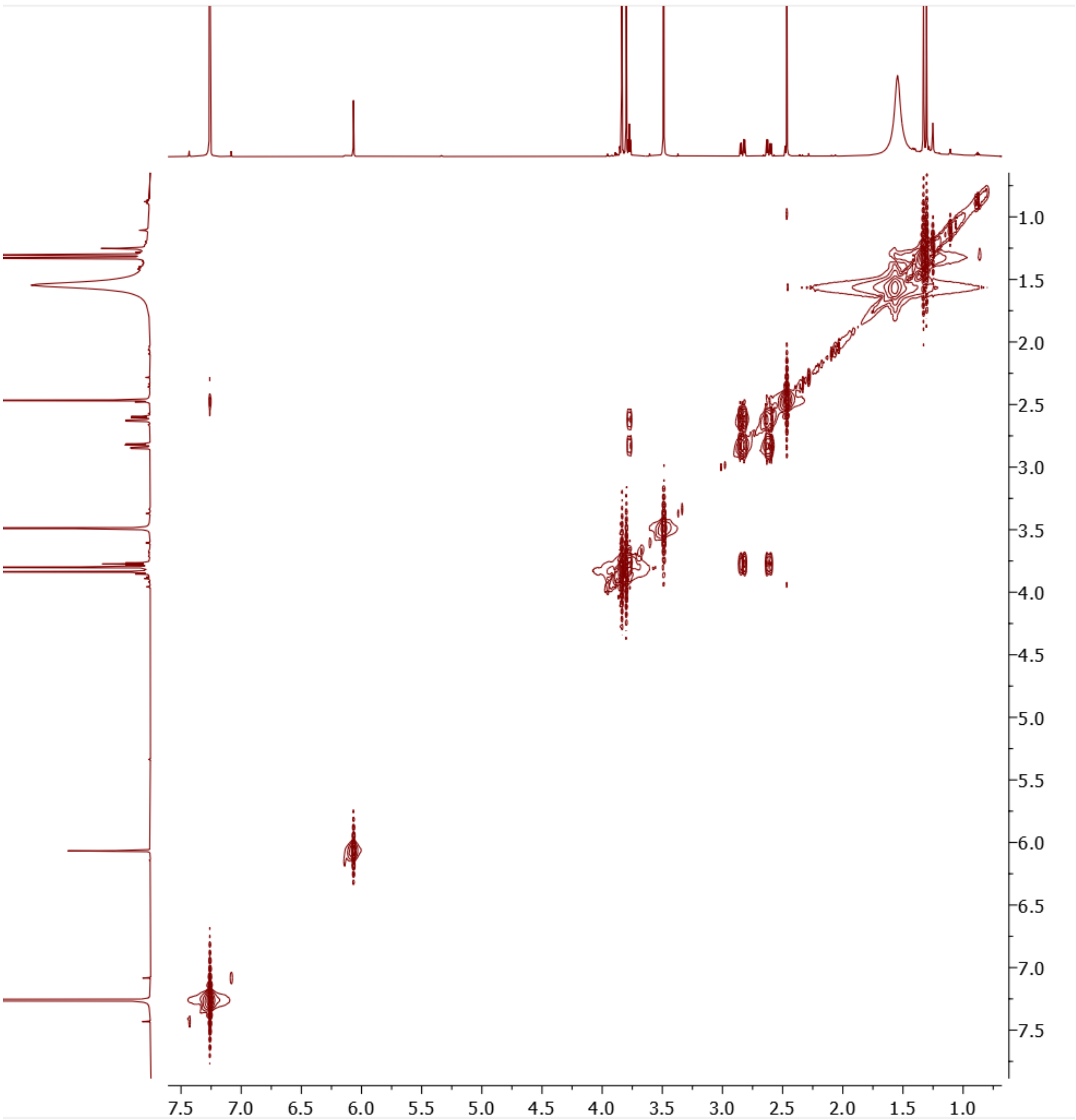

Figure S22. COSY (600 MHz, $CDCl_3$) spectrum of compound **4**.

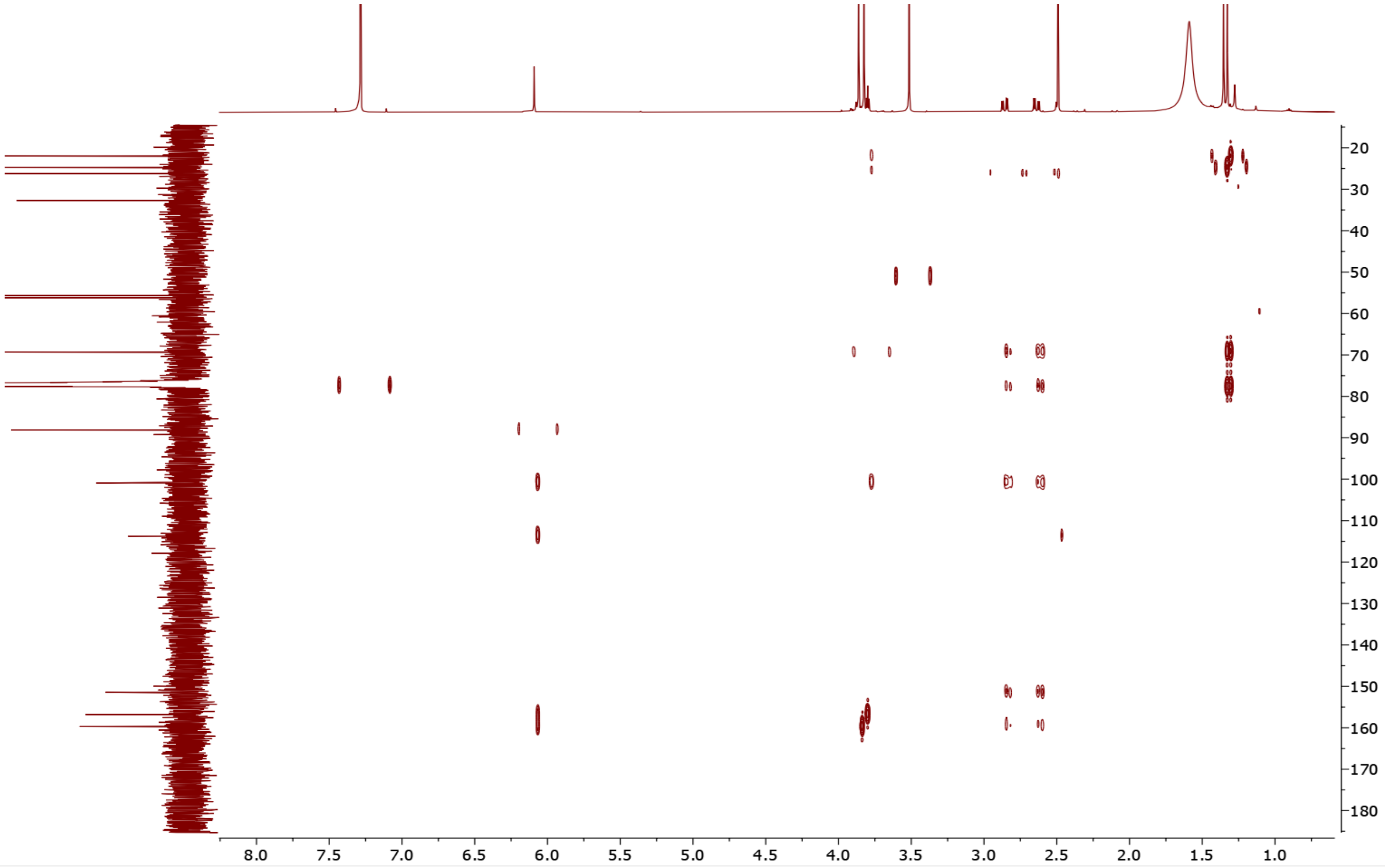

Figure S23. HSQC (600 MHz/150 MHz, $CDCl_3$) spectrum of compound **4**.

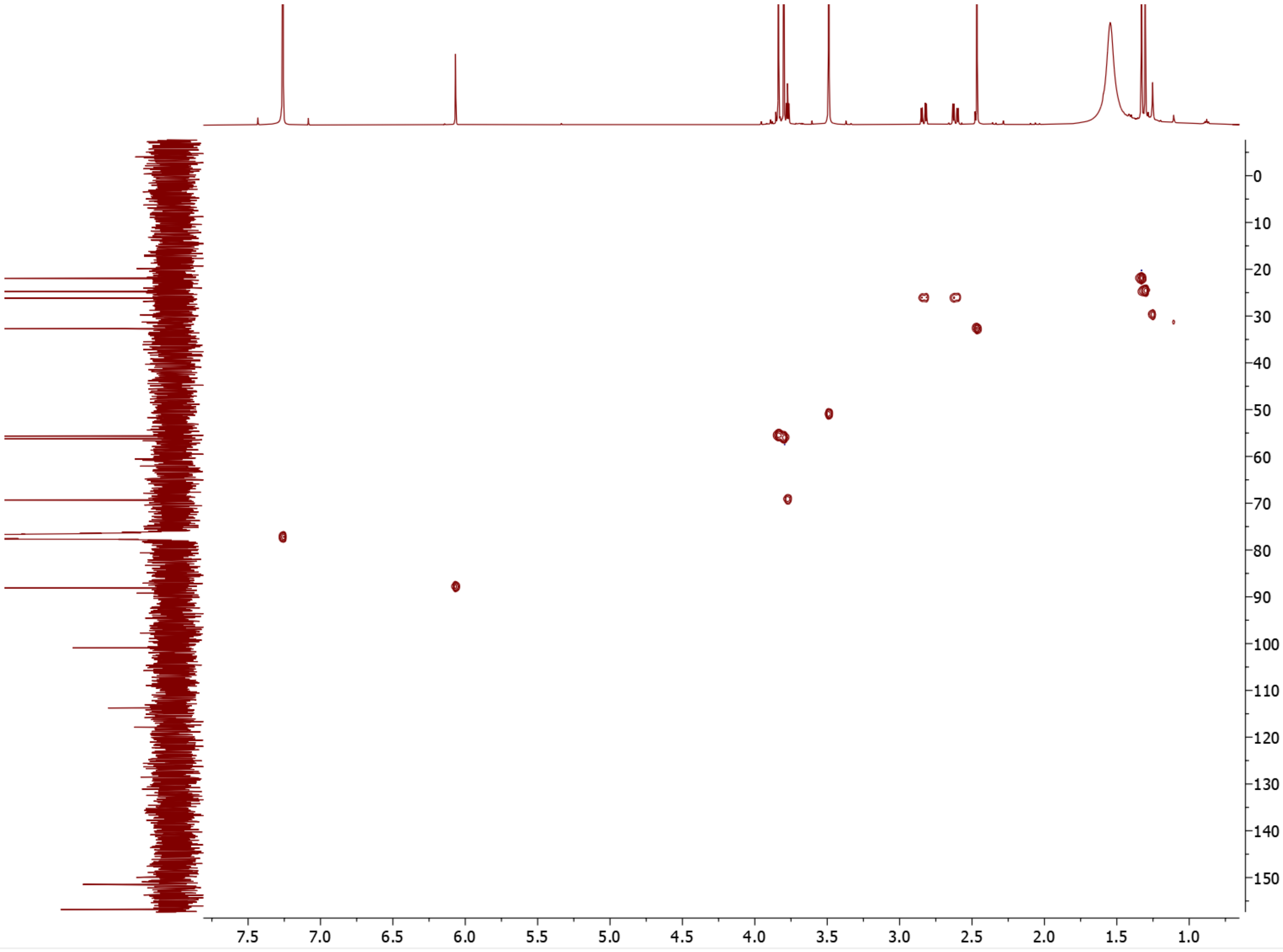

Figure S24. HMBC (600 MHz/150 MHz, $CDCl_3$) spectrum of compound **4**

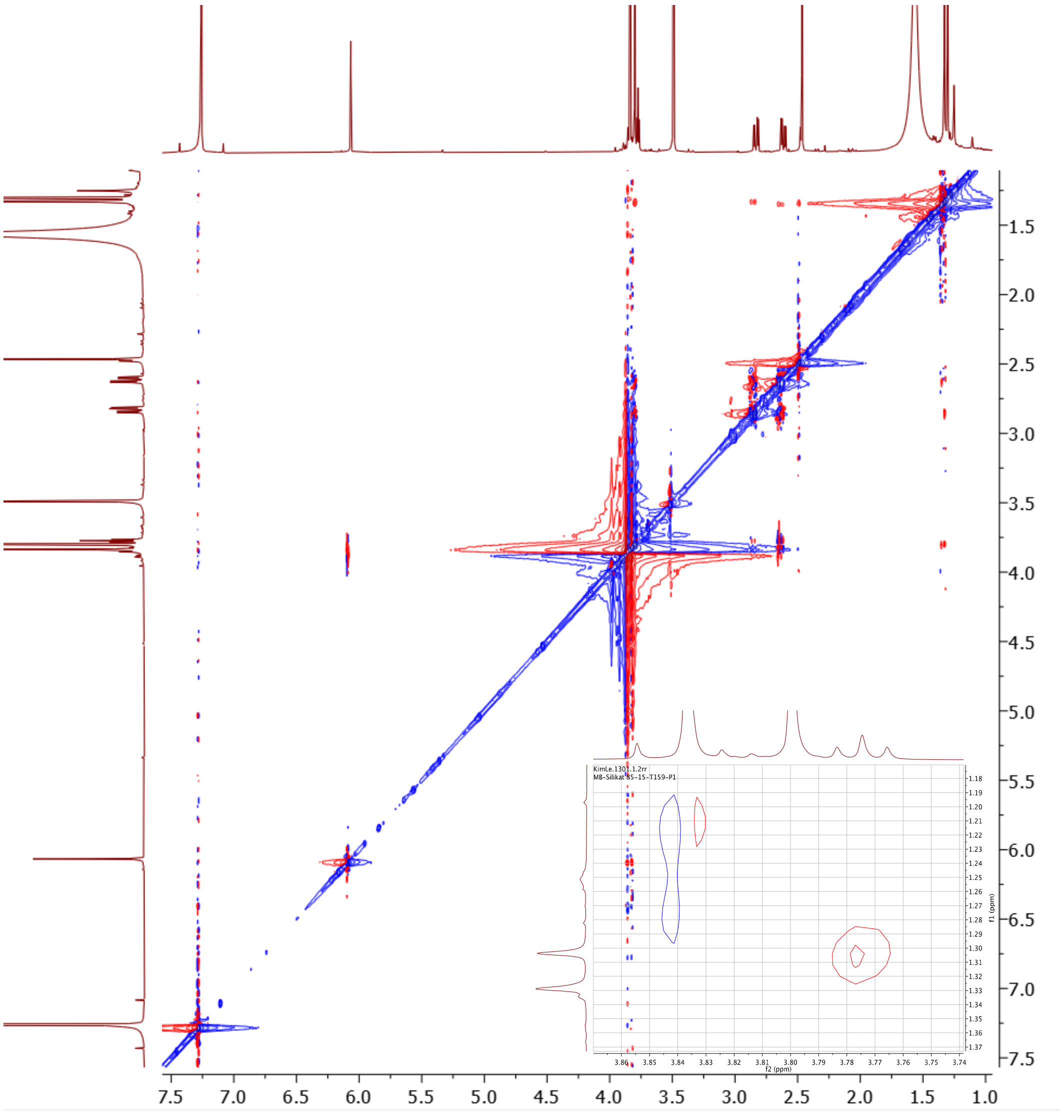

Figure S25. ROESY (600 MHz, $CDCl_3$) spectrum of compound **4**

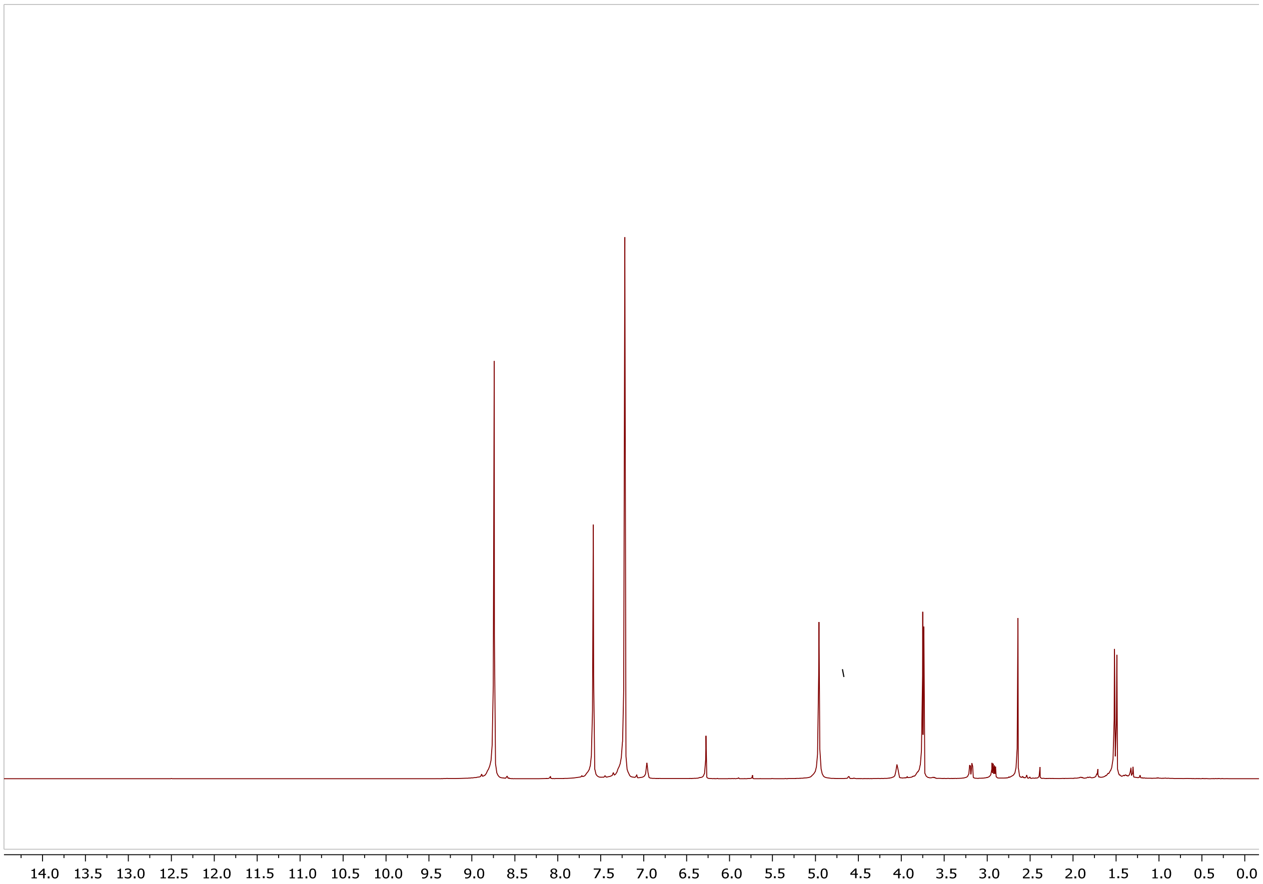

Figure S26. ^{1}H NMR (600 MHz, pyridine-d_5) spectrum of compound **4**

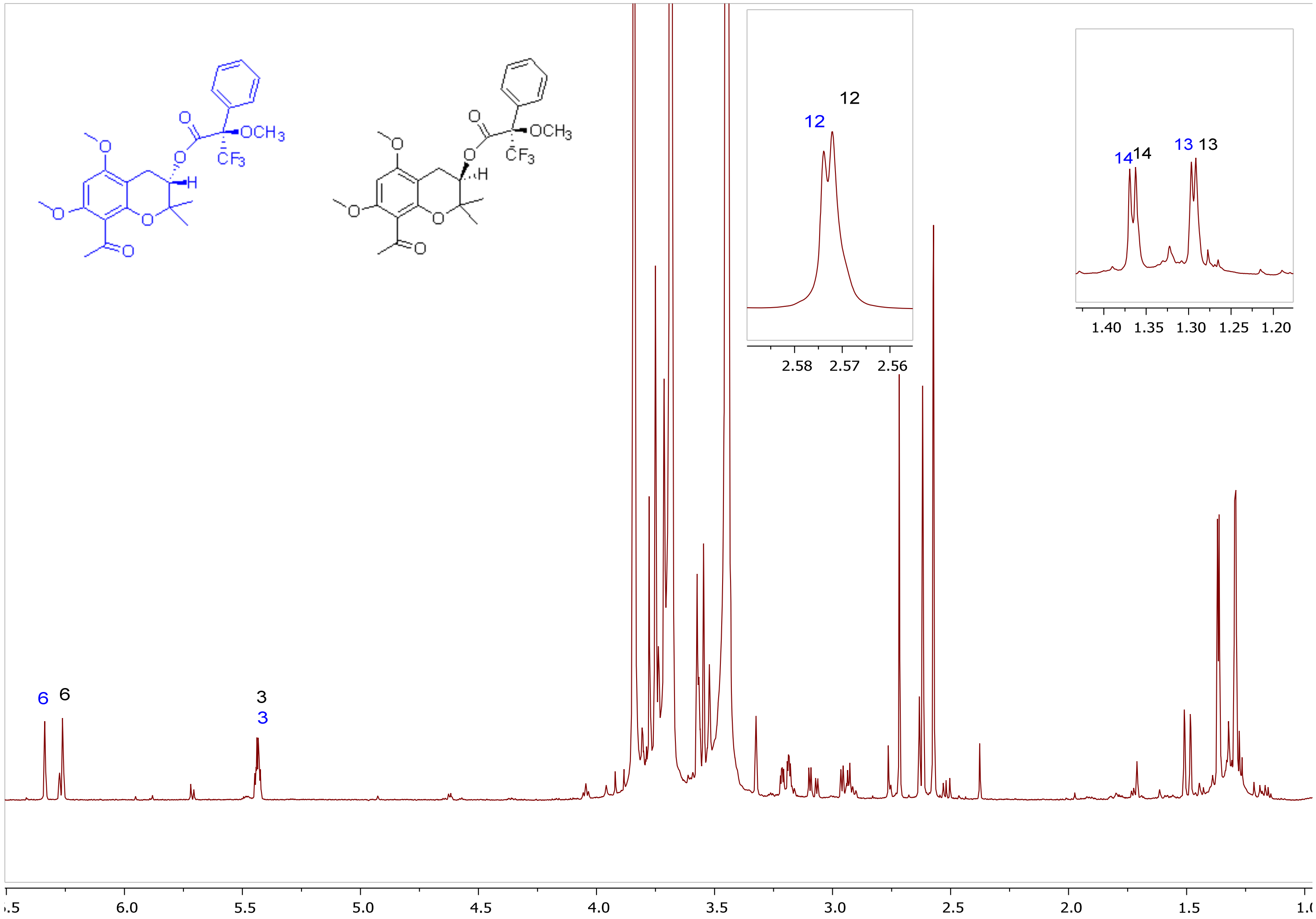

Figure S27. ^{1}HNMR spectra of **4** after reaction with (*S*)-MTPA reagent in pyridine-d_5

Acquisition Parameter

Source Type	ESI	Ion Polarity	Positive	Set Nebulizer	0.3 Bar
Focus	Not active	Set Capillary	4000 V	Set Dry Heater	180 °C
Scan Begin	50 m/z	Set End Plate Offset	-500 V	Set Dry Gas	4.0 l/min
Scan End	1500 m/z	Set Collision Cell RF	600.0 Vpp	Set Divert Valve	Source

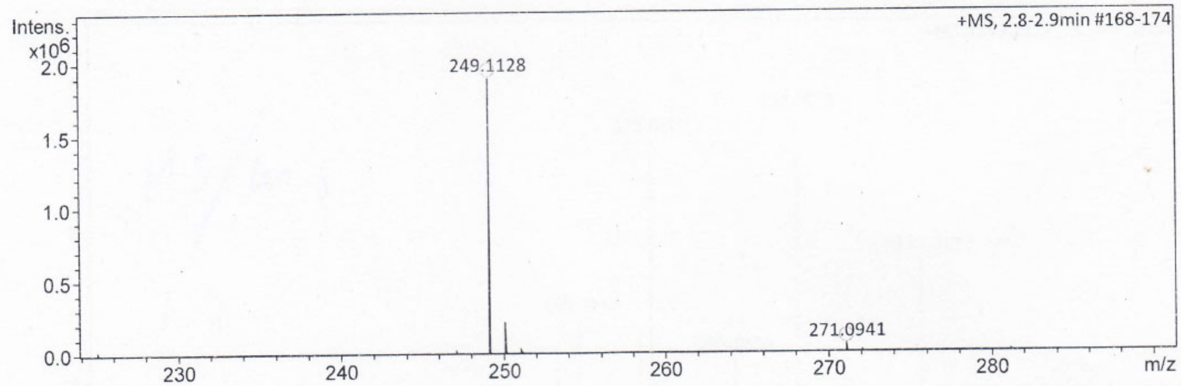

Meas. m/z	#	Ion Formula	m/z	err [ppm]	mSigma	# mSigma	Score	rdb	e⁻ Conf	N-Rule
249.1128	1	C14H17O4	249.1121	-2.5	23.6	1	100.00	6.5	even	ok
	2	C15H13N4	249.1135	2.9	36.9	2	68.84	11.5	even	ok
271.0941	1	C14H16NaO4	271.0941	-0.2	11.4	1	100.00	6.5	even	ok
	2	C15H12N4Na	271.0954	4.7	17.8	2	47.39	11.5	even	ok

Figure S28. HRESIMS of compound **5**.

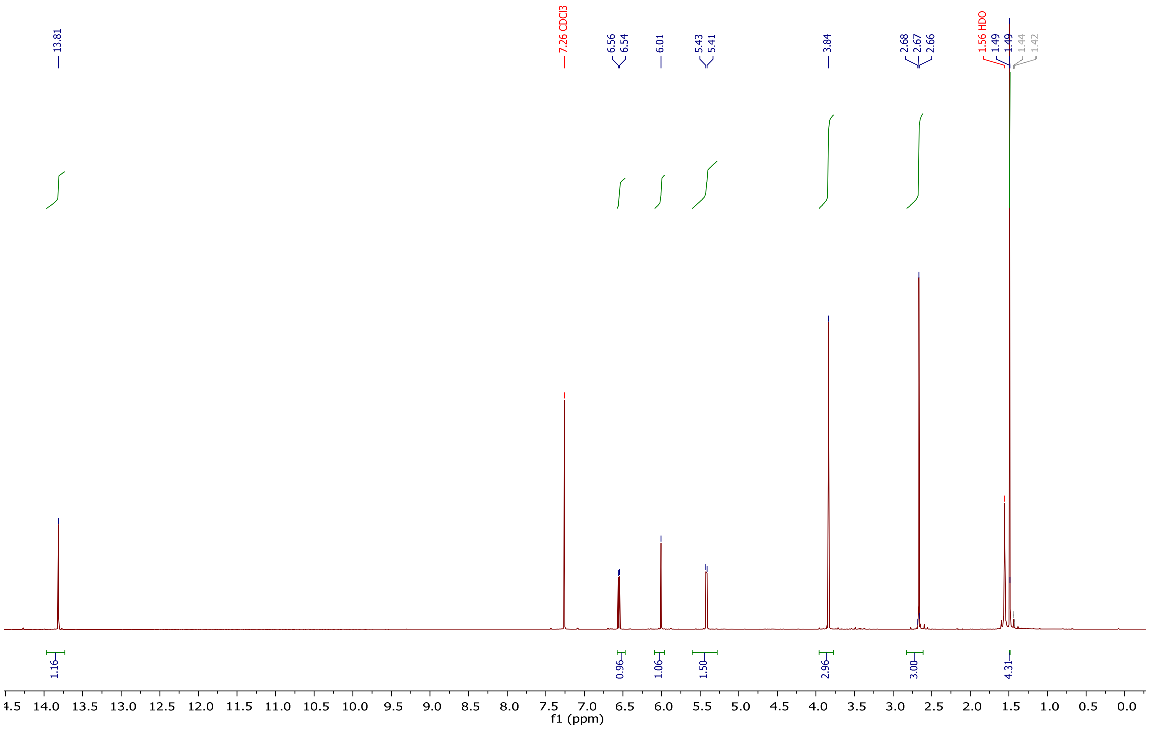

Figure S29. ^{1}H NMR (600 MHz, $CDCl_3$) spectrum of compound **5**.

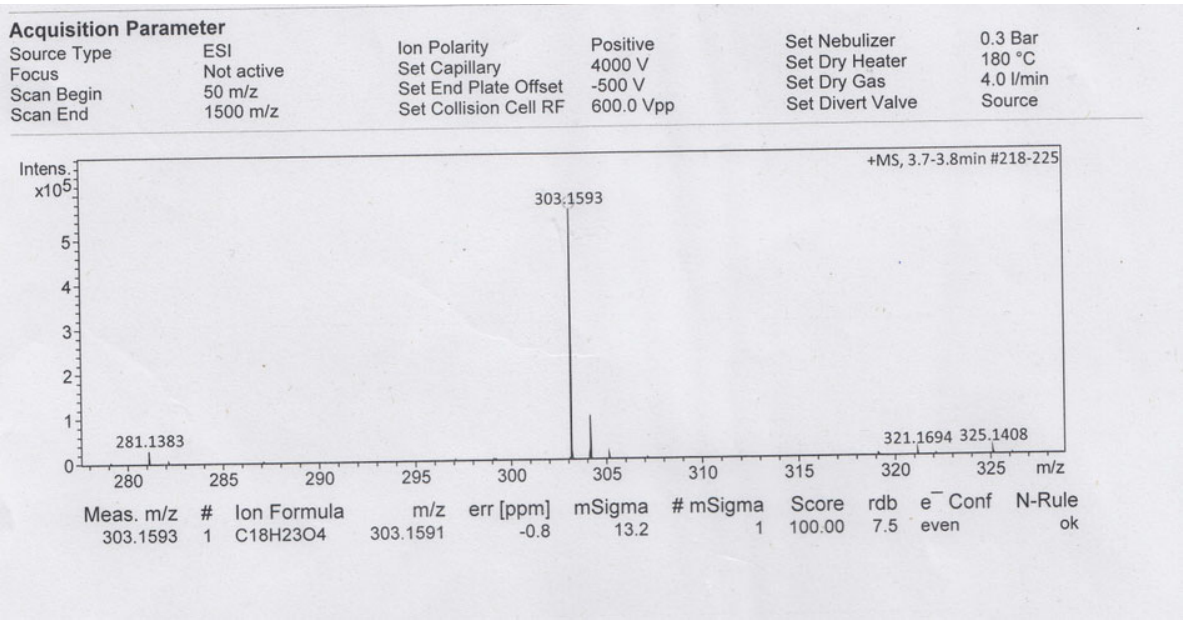

Figure S30. HRESIMS of compound **6+7**.

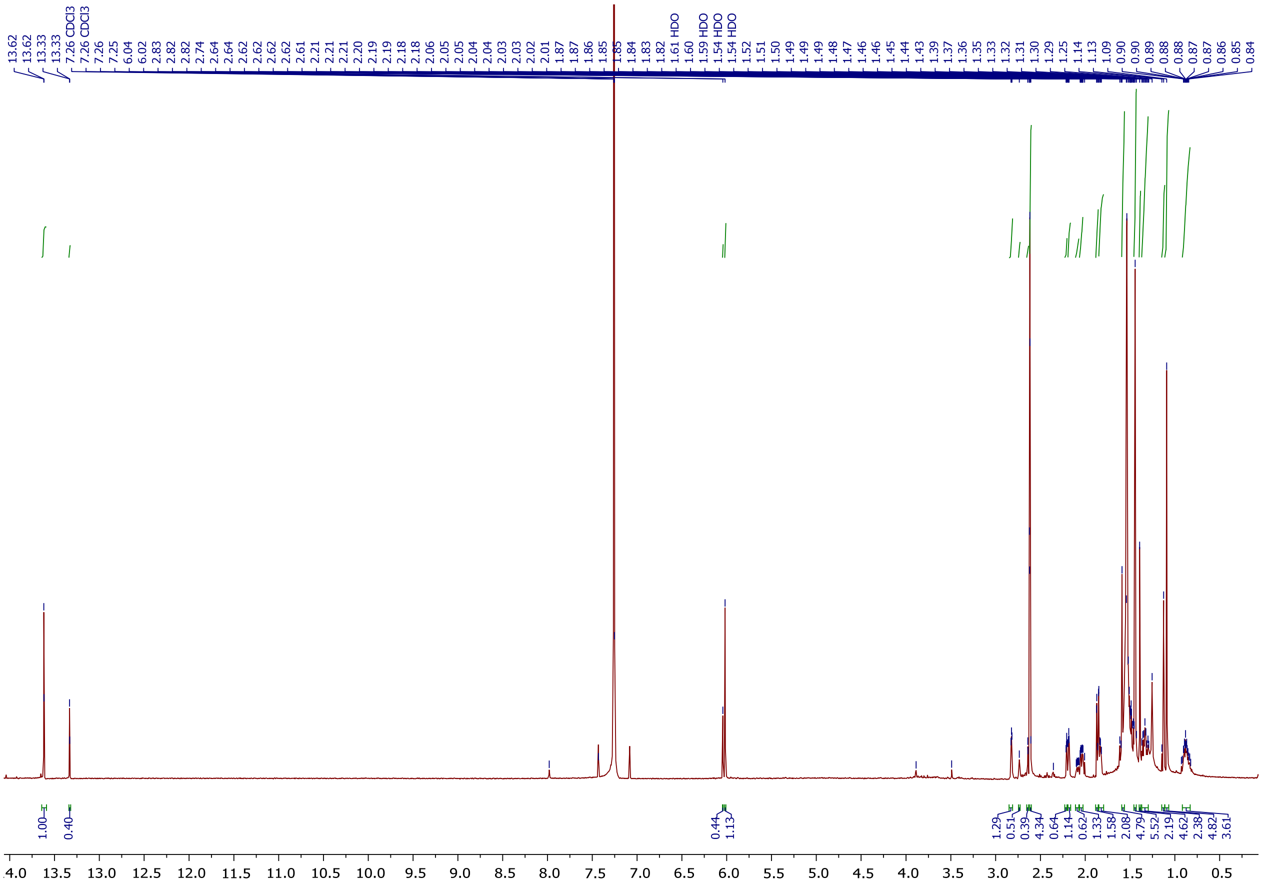

Figure S31.^{1}H NMR (600 MHz, $CDCl_3$) spectrum of compound **6+7**.

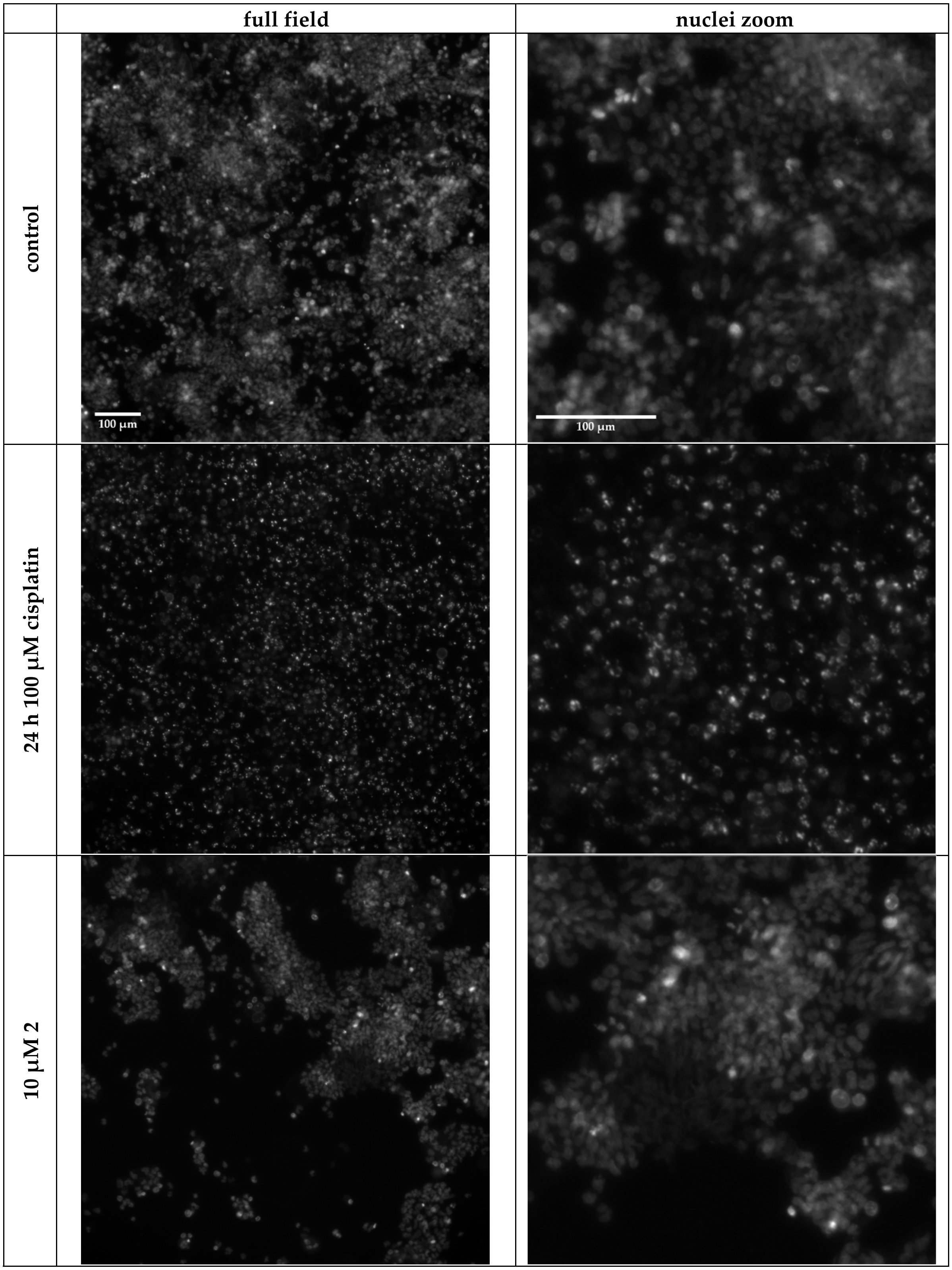
full field
nuclei zoom
control
24 h 100 μM cisplatin
10 μM 2
100 μm
100 μm

100 μM 2	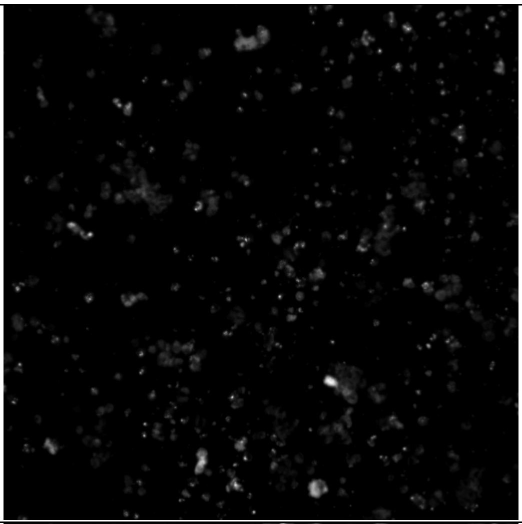	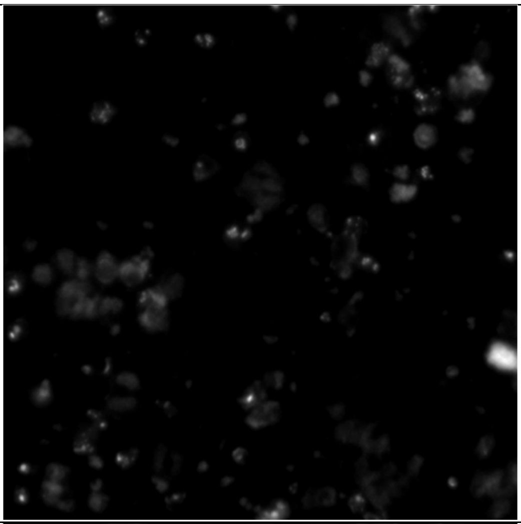
100 μM 3	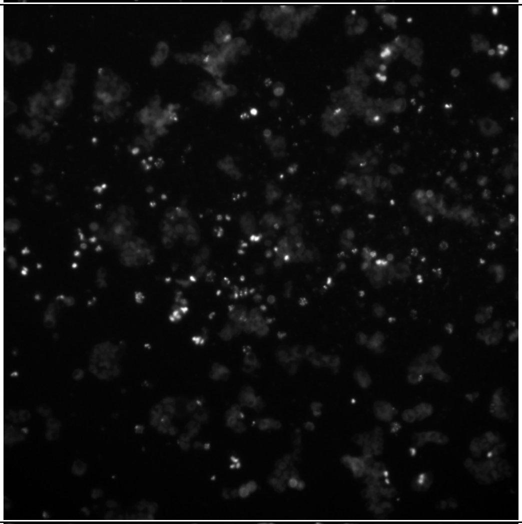	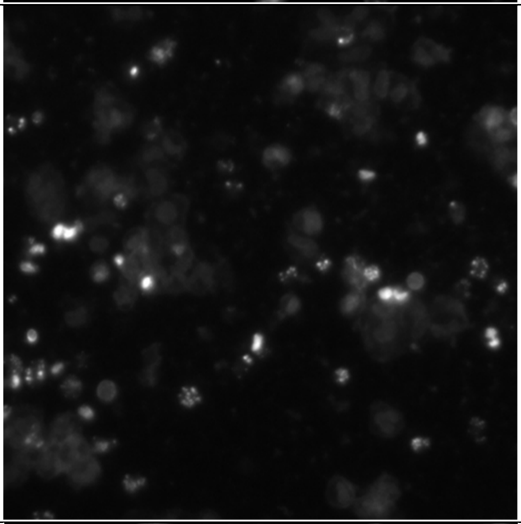
100 μM 4	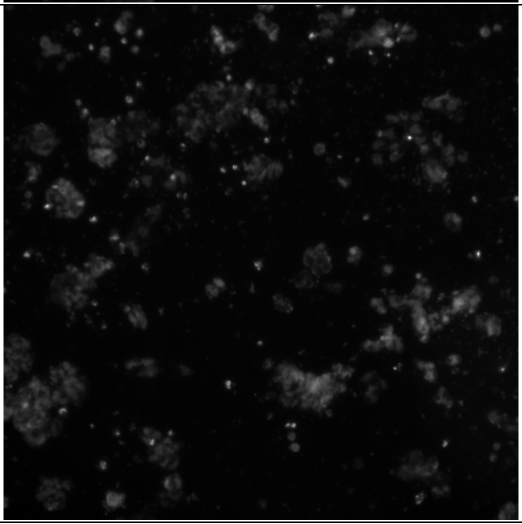	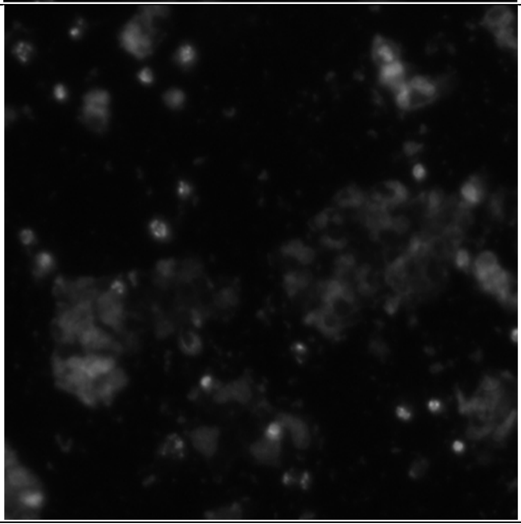

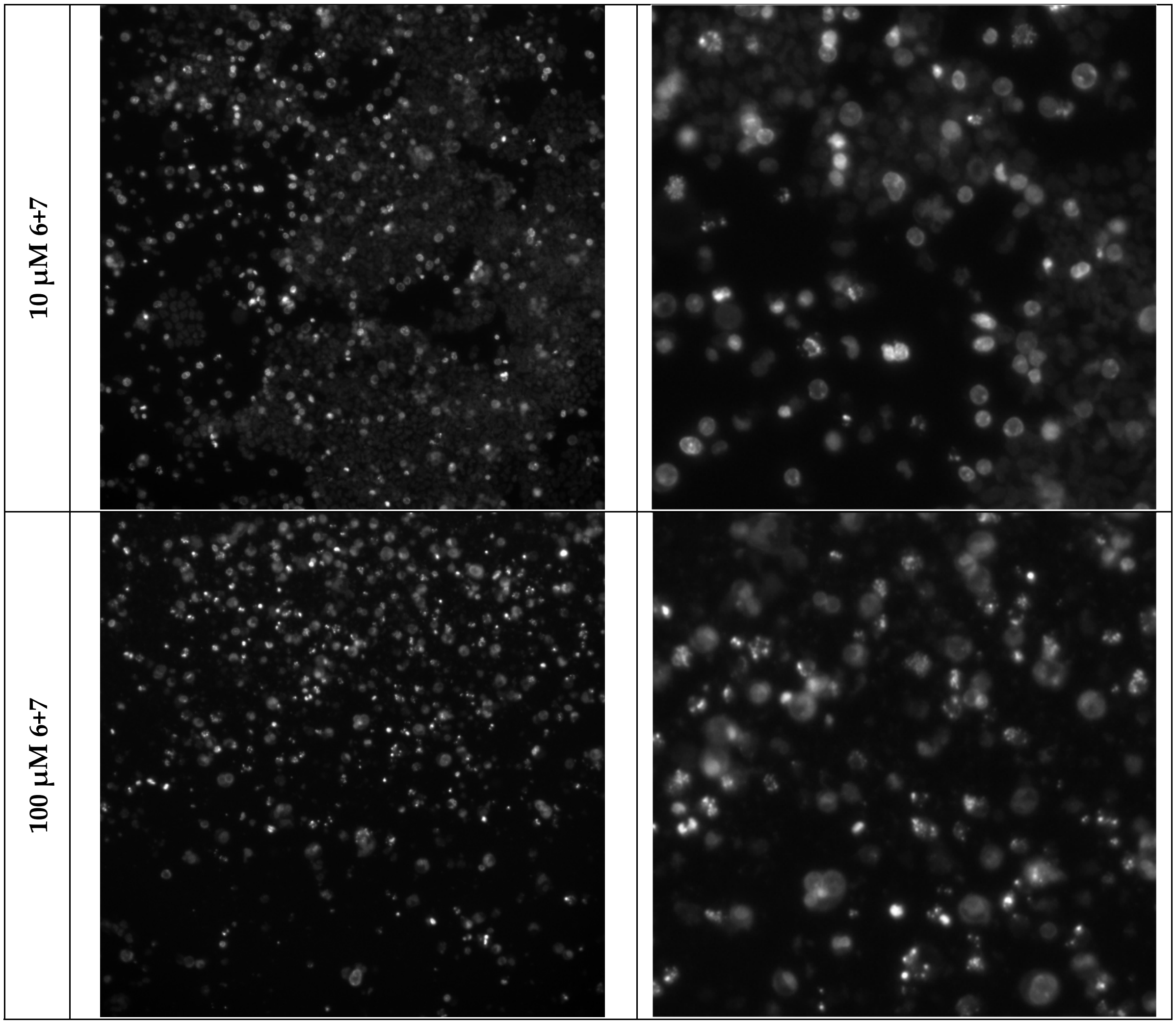

Representative fluorescent imaging pictures (10X magnification) for compounds with significant effects (corresponding to figure 5) are shown. Cells nuclei were stained with Hoechst-33342. A2780 cells were incubated with the compounds in the indicated concentrations for 72 h. Cell culture medium was added as a control for vehicle treated cells (“control”). A 24 h treatment with 100 μM cisplatin served as positive control. Scale bar in upper images (“control”) is 100 μm and applies to all images in the respective column of the figure.

Figure S32. Cytotoxic activity of compounds of *Melicope barbigera*

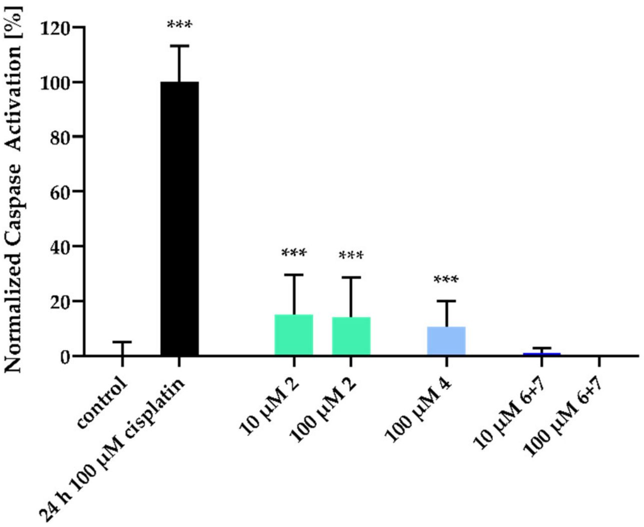

A2780 cells were incubated with the compounds in the indicated concentrations for 72 h. Cell culture medium was added as a control for vehicle treated cells ("control"). A 24 h treatment with 100 μM cisplatin served as positive control. To verify the involvement of caspases in the observed effects, 20 µM QVD was preincubated for 30 min prior to compound addition. No caspase 3/7-activation was obtained (data not shown). Data are the mean ± SD, n ≥ 3. Statistical analysis to compare the effects of compound and control was performed using t-test. For normalization, the value of the vehicle control was set to 0 % and the 24 h 100 μM cisplatin control was set to 100 %. Levels of significance: ns (p > 0.05); * (p ≤ 0.05); *** (p ≤ 0.001). Effect bars without annotation are ns.

Figure S33. Effects on caspase 3/7-activation of cytotoxic compounds of *Melicope barbigera*

Loci	A2780	A2780 (ECACC)
**D5S818**	11, 12	11, 12
**D16S539**	11, 13	11, 13
**vWA**	15, 16	15, 16
**D13S317**	12, 13	12, 13
**CSF1PO**	10, 11	10, 11
**TPOX**	8, 10	8, 10
**TH01**	6	6
**D21S11**	28	-
**D7S820**	10	10
**AMEL**	X	X

Shown are the results of the short tandem repeat (STR) analysis of the cell line A2780. The results were compared with the data of the cell bank ECACC and it can be stated that A2780 was successfully authenticated.

Table S1. Results of STR analysis of A2780
